# Metabolomic and metallomic profile differences between Veterans and Civilians with Pulmonary Sarcoidosis

**DOI:** 10.1038/s41598-019-56174-8

**Published:** 2019-12-20

**Authors:** Mohammad Mehdi Banoei, Isabella Iupe, Reza Dowlatabadi Bazaz, Michael Campos, Hans J. Vogel, Brent W. Winston, Mehdi Mirsaeidi

**Affiliations:** 1 Department of Critical Care, University of Calgary, Calgary, Canada; 2 Department of Medicine, University of Miami, Miami, FL USA; 3 Department of Biological Science, Bio-NMR-metabolomics Research center, University of Calgary, Calgary, Canada; 4 Departments of Critical Care Medicine, Medicine and Biochemistry and Molecular Biology, University of Calgary, Calgary, Alberta Canada; 5 Section of Pulmonary, Miami VA Healthcare System, Miami, FL USA; 6 Division of Pulmonary and Critical Care, University of Miami, Miami, FL USA

**Keywords:** Predictive markers, Translational research

## Abstract

Sarcoidosis is a disorder characterized by granulomatous inflammation of unclear etiology. In this study we evaluated whether veterans with sarcoidosis exhibited different plasma metabolomic and metallomic profiles compared with civilians with sarcoidosis. A case control study was performed on veteran and civilian patients with confirmed sarcoidosis. Proton nuclear magnetic resonance spectroscopy (^1^H NMR), hydrophilic interaction liquid chromatography mass spectrometry (HILIC-MS) and inductively coupled plasma mass spectrometry (ICP-MS) were applied to quantify metabolites and metal elements in plasma samples. Our results revealed that the veterans with sarcoidosis significantly differed from civilians, according to metabolic and metallomics profiles. Moreover, the results showed that veterans with sarcoidosis and veterans with COPD were similar to each other in metabolomics and metallomics profiles. This study suggests the important role of environmental risk factors in the development of different molecular phenotypic responses of sarcoidosis. In addition, this study suggests that sarcoidosis in veterans may be an occupational disease.

## Introduction

Sarcoidosis is a granulomatous entity of unknown etiology. The incidence of sarcoidosis has been well studied in the American civilian population and ranges between 11 in 100,000 in Caucasians to up to 36 in 100,000 in African American populations^1^. Although not well studied, the incidence of sarcoidosis among veterans is estimated to be even higher^2^. Sarcoidosis has protean manifestations including ophthalmic, joint, skin, and liver involvements, but most often involves the lung, affecting almost 90% of cases. In some individuals it can lead to serious disability of affected organs such as pulmonary fibrosis, cirrhosis, blindness, and may be fatal. Unfortunately, there is no available noninvasive biomarker to facilitate the diagnosis nor predict progression of disease.

Metabolomics is a method to identify and measure small molecules – metabolites – which may provide a way to study and monitor disease progression. In addition, has the potential to differentiate various stages of a particular disorder, provide means for a more accurate diagnosis and possibly stratification of prognosis^3,4^. Two of the more commonly used analytical platforms for metabolomics studies are proton nuclear magnetic resonance (^1^H-NMR) spectroscopy, a very robust and consistent method, and hydrophilic interaction liquid chromatography mass spectrometry (HILIC-MS), an extremely sensitive method^5^. Metabolomics has helped to identify potential biomarkers for the diagnosis and prognosis of infectious and non-infectious respiratory conditions such as asthma^6^, chronic obstructive pulmonary disease (COPD)^7,8^, influenza^9^, pneumonia^10^, tuberculosis^11^, and acute respiratory distress syndrome (ARDS)^12,13^.

Metallomics targets various elements and metal ions that participate in many biological pathways in close relationship with proteins and metabolites^14^. Inductively coupled plasma-mass spectrometry (ICP-MS) is a highly sensitive technique used to detect and quantify elements in the periodic table in biological fluids^15^.

The veteran population comprise a particular group of subjects with a unique history of exposure to hazardous materials, including gun smoke, jet fuel, air pollution (burn pit smoke, dust) and occupational hazards (asbestos, lead)^16^. Because of this, we sought to evaluate whether veterans with sarcoidosis exhibit different metabolomics and metallomics profiles compared with civilians with sarcoidosis. Furthermore, we evaluated whether veterans with sarcoidosis have a different metabolomic and metallomic profile compared to other veterans with a non-sarcoidosis pulmonary condition, (COPD).

## Methods

### Patient enrollment

We performed a case-control study on veterans (n = 13) and civilians (n = 30) with confirmed pulmonary sarcoidosis. Sarcoidosis was defined as the presence of clinical signs and symptoms of pulmonary sarcoidosis or the presence or history of bilateral hilar lymphadenopathy, along with biopsy-proven sarcoid-like granulomas in pulmonary samples, with the exclusion of other granulomatous conditions, including mycobacterial infection.

Airflow obstruction was defined per American Thoracic Society and European Respiratory Society (ATS/ERS) guidelines with an FEV1/FVC ratio less than lower limit of normal (24). In order to further analyze the metabolomic profiles of veterans without sarcoidosis and with similar exposure history, we randomly selected 35 veterans with COPD as controls, matched by race, gender, and deployment history. Civilians were recruited from the University of Miami Sarcoidosis Program and veterans from the Miami Veterans Administration Sarcoidosis Program.

This retrospective study was conducted in accordance with Helsinki Declaration and the study was been approved by the University of Miami’s Institutional Review Board (No.20150612). Written informed consent was obtained from all participants.

### ^1^H-NMR spectroscopy and metabolites profiling

Plasma samples were obtained from all patients and analyzed by ^1^H-NMR spectroscopy. Details of sample preparation have been described in the Supplementary Appendix (Supp. 1). One dimensional ^1^H-NMR was performed using a 600 MHz Bruker Ultrashield Plus ^1^H-NMR spectrometer (Bruker BioSpin Ltd., Canada). Details of the ^1^H-NMR analysis have previously published^9^ and provided in the Supp. 1. To quantify the metabolite concentrations, DSS (4,4-dimethyl-4-silapentane-1-sulfonic acid) was used as an internal standard. Multivariate statistical analysis models were developed to identify metabolites involved in the discrimination. ChenomX 7.1 was used to identify and quantify metabolites^17^.

### HILIC-MS and metabolites profiling

Plasma samples were also analyzed by liquid chromatography mass spectroscopy (LC-MS) using the Q Exactive HF Hybrid Quadrupole-Orbitrap Mass Spectrometer, Thermo-Fisher). Chromatography was performed using a 2.1 mm × 100 mm long Syncronis HILIC (thermos-Fisher) LC column packed in-house with 3 µm porous Hypercarb particles.

The elution gradient of solvent B (%) (acetonitrile with 0.1% formic acid) over time was: 95% for 2 min, 85%–95% for 5 min, 5–80% for 3 min, and 5% for 5 min, 5–95% for 2 min and then held at 95% for last 3 min against solvent A (20 mM ammonium formate pH 3.0 in H_2_O). MS conditions were as follows: HESI-II temperature 325 °C, auxiliary gas flow 10 units, sheath gas flow 25, spray voltage ± 2.50 kV, capillary temperature 275 °C, and S-lens RF level 60%, auxiliary gas heater temperature 275 °C for negative ion mode. To acquire mass spectra, mass scan parameters were set up as follows: runtime 20 mins, full MS scan type, resolution 240,000, AGC target 3e6, maximum IT 200 ms and scan range 70–1000 m/z. Maven software, an open source software, was used for processing metabolomics data obtained by LC-MS^18^.

### ICP-MS analysis of metallome profiles

The plasma metallome was assessed using inductively coupled plasma mass spectrometry (ICP-MS). Details of the sample preparation is also described in the Supp. 1. ICP-MS analysis was performed using a PlasmaQuant® MS Elite (Analytik Jena, Jena, Germany) spectrometer on plasma samples. A standard Seronorm^TM^ serum samples was used as quality control samples and provided a way to translate the elemental count of the ICP-MS to semi quantitative. Integrated collision reaction cell (iCRC) mode was applied for analysis of plasma using a single continuous method. To attenuate all polyatomic interference, hydrogen gas was added to the iCRC skimmer. Both iCRC and non-iCRC modes were applied for analysis, thus the isotopes (different forms of an element with ‘different number of neutrons) ^51^V, ^75^As, ^78^Se and ^90^Zr were quantified by both iCRC mode and non-iCRC mode. ^24^Mg, ^56^Fe and ^51^Cr were quantified only in iCRC mode and non-iCRC mode was used for all the remaining isotopes.

### Data analysis

Both univariate and multivariate data analyses were applied to the extracted information from the metabolomic datasets. Univariate analyses such as, ANOVA and T-test were used as complementary methods to the multivariate analysis in order to provide useful information on metabolomics profiles and on metabolite individually. MetaboAnalyst^19^ and MetaBox^20^ were used for univariate data analyses. Data were not preprocessed for the univariate analyses.

Multivariate data analysis was applied to reduce the complexity of metabolomics data and for data mining. Principal component analysis (PCA) was performed to find outliers, trends, and similarities, using datasets derived from the plasma samples for the evaluation of interrelations and groupings of metabolomics data between veteran and civilians with pulmonary sarcoidosis and veterans with COPD.

Partial least square-discriminant analysis (PLS-DA) and orthogonal partial least square-discriminant analysis (OPLS-DA) are two supervised multivariate data analyses methods for classification and discrimination between groups by maximizing separation using most differentiating variables responsible for class discriminations. PLS-Da and OPLS-DA were used to separate different populations and to establish metabolomic profiles for each population based on the most differentiating metabolites. Q^2^Y (goodness of validation) and cross validation ANOVA (CV-ANOVA) parameters were considered for predictability and significance of separation of study populations using metabolomics profiles^21,22^. Q^2^Y > 0.3 and 0.5 were evaluated as acceptable and good models for human samples. P value ≤ 0.05 was considered a significant model. The highest Q^2^Y was considered in choosing the OPLS-DA model. S-plot and coefficient plot were applied to find the most significant metabolites that contributed to the separation as potential variables in the study. S-plot was used to extract the putative biomarkers, that have both high reliability and magnitude. Compounds with covariance >0.1 were considered important metabolites^23^. SIMCA-P v15.0.2 (Umetrics AB, Umeå, Sweden) and MetaboAnalyst 3.0 software were used for multivariate analyses. Data were normalized (median), transformed (log) and auto-scaled for multivariate analyses.

### Prediction test

Prediction test was used to obtain sensitivity, specificity and area under the curve for receiver operating (AUROC) parameters. The prediction test was carried out by splitting the population of the study into a training and a predicting set. The predicting group was created by randomly selecting 25% of the samples. A misclassification table was obtained for all discriminant analysis models to measure sensitivity and specificity using SIMCA-P v15.0.2 software. AUROC was calculated using Graph Pad Prism v 3.03.

### Pathway analysis

Parallel pathway analyses were performed using MetaboAnalyst 3.0^24^, a free web-based tool, and Cytoscape 3.6.0^25^ using selected discriminative metabolites in the separation of veteran sarcoidosis from civilian cohorts. Reactome, an open-source, open access, pathway database was also used to explore biochemical networks^26^. The list of discriminative metabolites was chosen according to the best OPLS-DA model which had higher predictability (Q^2^Y).

## Results

### Patient characteristics

Of the 78 subjects who were consented for the study, 43 had sarcoidosis (13 veterans and 30 civilians) and 35 were veterans with COPD. Demographic characteristics are summarized in Table 1. Out of the 43 subjects enrolled with sarcoidosis, 10 subjects (33%) in the civilian group and 1 subject (8%) in the veteran group were female (P = 0.107). There were no significant differences in age, race or lung function between participants. The difference in the mean (SD) CPI score of civilian and veteran subjects were 19.1 (21.3) and 29.2 (18.8), respectively (P = 0.0148).

**Table 1:** Demographic and clinical characteristics in the veteran and civilian populations studied.

Variables	Sarcoidosis		P-value	COPD	P-value^*^
	Veterans N = 13 (%)	Civilian N = 30 (%)		Veterans N = 35 (%)	
Race: African American	3(23)	6(20)	0.820	12(34)	0.460
Gender: Female	1(9)	10(33)	0.107	2(6)	0.802
Presence of airflow obstruction	3(23)	8(27)	0.804	N/A	N/A
Taking Prednisone >20 mg/d	4(31)	3(10)	0.105	N/A	N/A
Taking 2^nd^ line antisarcoidosis medications	5(39)	15(50)	0.488	N/A	N/A
Extrapulmonary sarcoidosis	7(59)	19(63)	0.560	N/A	N/A
On Anti-TNF*-α therapy*	2(15)	4(13)	0.859	N/A	N/A
Congestive heart failure	0	2(7)	0.870	4(11)	0.498
Diabetes	4(31)	8(27)	0.783	5(14)	0.203
Chronic kidney disease	1(8)	1(3)	0.544	3(8.6)	0.922
Hypertension	9(69)	13(43)	0.126	23(66)	0.818
Hyperlipidemia	8(62)	10(33)	0.091	0	< 0.0001
Coronary artery disease	1(8)	1(3)	0.544	8(23)	0.256
FVC < 70%	3(23)	6(20)	0.819	N/A	N/A
DLCO (MEAN ± SD)	67.6 ± 23.3	68.2 ± 23.1	0.9	51.7 ± 14.6	0.001
CPI (mean, SD)	29(21.3)	19.1(21.3)	0.015	N/A	N/A
6-min walk distance (m) (MEAN ± SD)	310 ± 128	259 ± 159	0.52	330 ± 108	0.28
Scadding criteria (based on chest images)					
Stage I	4 (31)	10 (33)	NS	N/A	N/A
Stage II	2(15)	7 (23)	NS	N/A	N/A
Stage III	3(23)	5 (17)	NS	N/A	N/A
Stage IV	4(31)	8 (27)	NS	N/A	N/A
mMRC (MEAN ± SD)	NA	NA	NA	1.87 ± 1	N/A
Deployment history					
Caribbean	0	N/A	N/A	1(3)	0.541
Europe	0	N/A	N/A	8(22)	0.065
Persian Gulf	4(31)	N/A	N/A	1(3)	0.004
South Asia	5(38)	N/A	N/A	11(31)	0.472
Never Deployed	4 (31)	N/A	N/A	15(41)	0.481

^*^P-value shows comparison between veterans with sarcoidosis and veterans with COPD. Scadding Stage IV: shows pulmonary fibrosis in sarcoidosis. A patient in COPD cohort was deployed to both Asia and Europe. SD is standard deviation, NS: non-significant. mMRC (Modified Medical Research Council) Dyspnea Scale. One patient has moderate pulmonary hypertension in COPD group and none of sarcoidosis subjects has pulmonary hypertension.

Among COPD subjects, the mean (SD) age was 63.5 (4.7), which was significantly older than veterans with sarcoidosis (P = 0.038). However, there was no difference in race and gender between these two groups.

### Metabolomics study

#### Metabolite Identification

Using ChenomX ^1^H-NMR Suite 7.1^27^, one dimensional ^1^H-NMR analysis resulted in the identification of 55 metabolites including sugars, organic acids, amino acids, and volatile organic compounds. Using the Maven software, 103 variables were identified in a targeted approach to the HILIC-MS analysis and consisted of sugars, amino acids, organic acids, acylcarnitines and their derivatives.

### Principal component analysis (PCA) clearly showed clustering between veteran and civilian sarcoidosis and COPD control

Unsupervised PCA demonstrated clustering among the three groups using the 55 and 103 metabolites detected by ^1^H-NMR and HILIC-MS, respectively (Fig. S1A,B). However, PCA showed a a higher degree of separation between veteran and civilian sarcoidosis cohorts reflecting two distinct metabolomic profiles (Fig. S2A,B). The 6-component PCA models had an R^2^X = 0.483 and 0.456 for ^1^H-NMR and HILIC-MS, respectively, demonstrating a relative high variation of metabolites among the samples. Further analysis revealed very good clustering between civilian sarcoidosis and veterans with COPD, whereas the two veteran cohorts were roughly clustered (Figs. S3 and S4).

### Veterans with sarcoidosis have a different metabolomic profile compared to civilians with sarcoidosis

**Figure 1:**
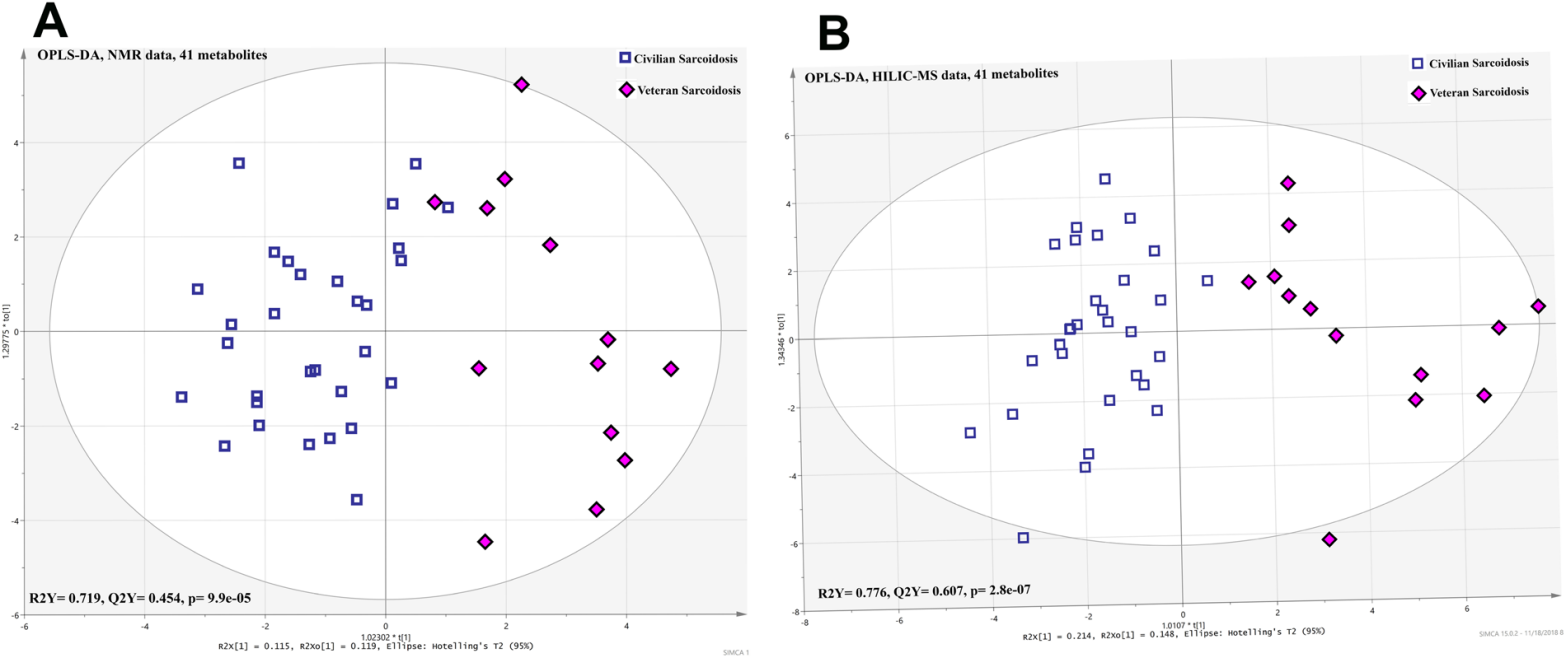
Shows OPLS-DA generated from ^1^H-NMR (**A**) and HILIC-MS (**B**) data, demonstrating a significant difference between the veteran and civilian subjects with pulmonary sarcoidosis with a higher predictive power for HILIC-MS (Q^2^Y = 0.607, p = 2.8 × 10^−7^) than ^1^H-NMR spectroscopy (Q^2^Y = 0.454, p = 9.9 × 10^−5^).

Multivariate data analysis of metabolomic profiles using OPLS-DA detected that the veteran sarcoidosis cohort was significantly different from the civilian sarcoidosis cohort. The results provided a highly predictive and significant model to separate the two cohorts particularly for HILIC-MS (Q^2^Y = 0.607, p = 2.8 × 10^−7^) than ^1^H-NMR spectroscopy (Q^2^Y = 0.454, p = 9.9 × 10^−5^) (Fig. 1A,B). Forty-one metabolites contributed to the differentiation of metabolic profiles between civilians and veterans with sarcoidosis for each of the two analytical approaches (Fig. S5A,B). Six metabolites were detected in both HILIC-MS and ^1^H-NMR to be significantly different between civilian and veteran sarcoidosis subjects: arginine, glutamine, creatinine, glycine, taurine, and methionine.

The performances of the discriminative models are summarized in Table 2. Most differentiating metabolites were selected for the OPLS-DA models to obtain higher predictability (Q^2^Y) using a variable importance in projection (VIP) approach. S-plot revealed that taurine, sucrose, n-methyl-d-aspartic acid, ll-2–6-diaminoheptanedioate, hypoxanthine, ethanolamine, alpha-hydroxyisobutyric acid, sn-glycerol 3-phosphate, 2-oxoisocaporate, 4-hydroxybutyrate, acetone histidine, isoleucine, isopropanol, methionine and beta-alanine were the most plausible biomarkers to differentiate between the two sarcoidosis cohorts in both datasets (Fig. S6A,B). This study also showed that a targeted analysis using HILIC-MS could be a better discriminative method because of its higher Q^2^ and the fact that includes all 103 identified metabolites (Fig. S7). Permutation test confirmed the validation of R^2^ and Q^2^ values of each predictive model (OPLS-DA) using 200 repetitions (Fig. S8).

**Table 2:** Summary of all OPLS-DA analyses (discriminative models) for H-NMR spectroscopy, LC-MS and ICP-MS analyses.

OPLS-DA model		R^2^Y	Q^2^Y	P value	Sensitivity	Specificity	AUROC	#Metabolites/elements*
NMR	Civilian vs. Veteran	**0.719**	**0.454**	**9.91**_**e**_**−005**	**100**	**100**	**1.0**	**41**
	Civilian vs. COPD	**0.898**	**0.713**	**4.62**_**e**_**−014**	**100**	**98**	**1.0**	**55**
	Veteran vs. COPD	**0.576**	**0.408**	**0.00012**	**86**	**76**	**0.90**	**13**
LC-MS	Civilian vs. Veteran	**0.776**	**0.607**	**2.86**_**e**_**−007**	**100**	**100**	**1.0**	**41**
	Civilian vs. COPD	**0.845**	**0.797**	**9.94**_**e**_**−20**	**100**	**100**	**1.0**	**26**
	Veteran vs. COPD	**0.663**	**0.46**	**0.0001**	**95**	**100**	**0.98**	**29**
ICP-MS	Civilian vs. Veteran	**0.654**	**0.555**	**2.97**_**e**_**−005**	**91**	**90**	**0.95**	17*
	Civilian vs. COPD	**0.918**	**0.838**	**2.89**_**e**_**−019**	**95**	**95**	**0.97**	57*
	Veteran vs. COPD	**0.639**	**0.458**	**0.0002**	**86**	**84**	**0.91**	29*

NMR: Nuclear magnetic resonance, LC-MS: Liquid chromatography–mass spectrometry, ICP-MS: Inductively coupled plasma mass spectrometry, R^2^Y: It is used to evaluate the model with showing the percentage of all NMR, LC-MS, and ICP-MS response variables explained by the model. Q^2^Y: It is used to evaluate the model with showing the percentage of all observation predicted by the model. AUROC: The Area Under the curve of the Receiver Operating Characteristic. *: Shows elements.

**Figure 2:**
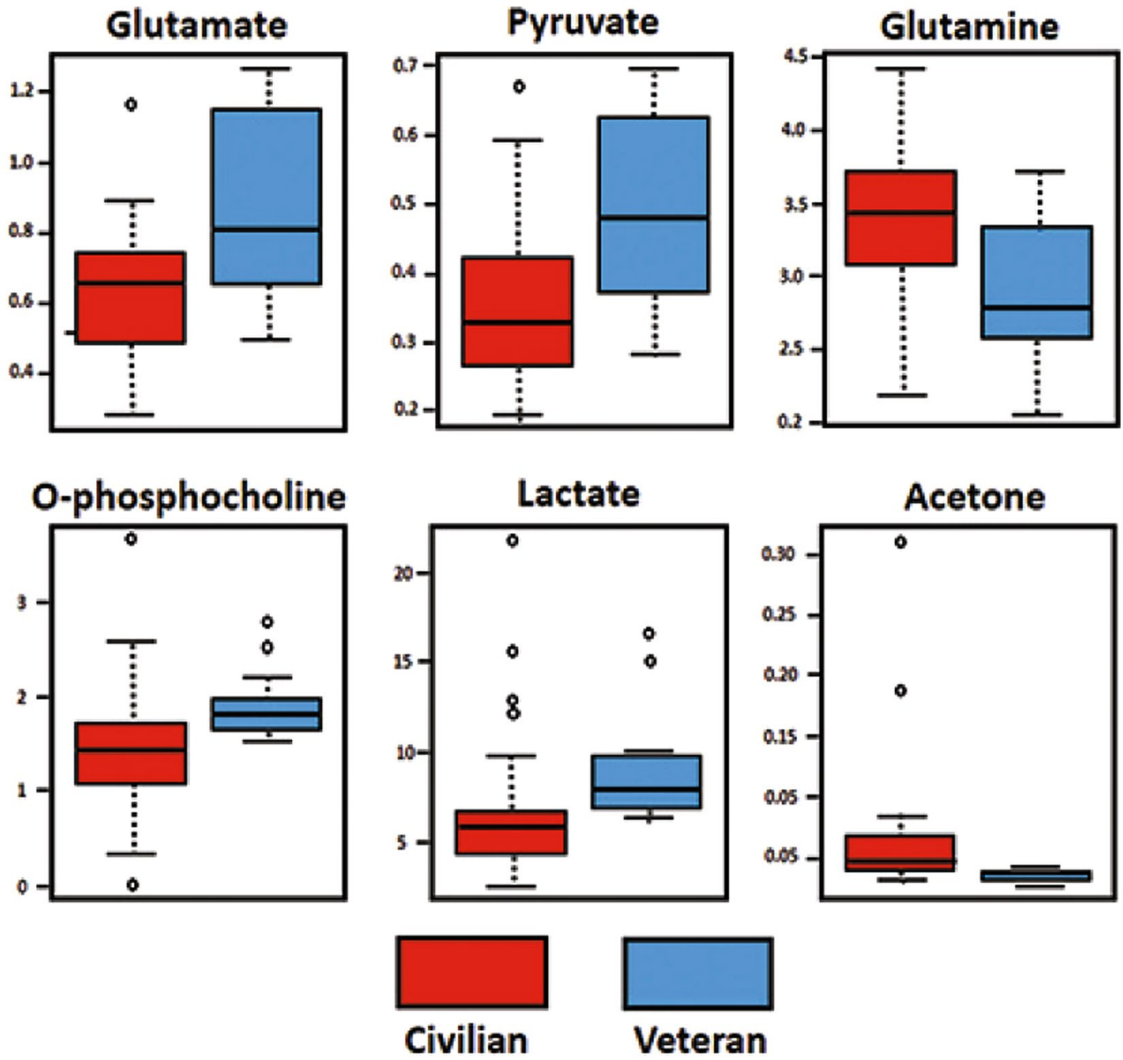
Shows 6 metabolites differentially expressed (p < 0.05), with significant FDR (p < 0.05) between the veteran and civilian sarcoidosis cohorts using the ^1^H-NMR dataset.

**Figure 3:**
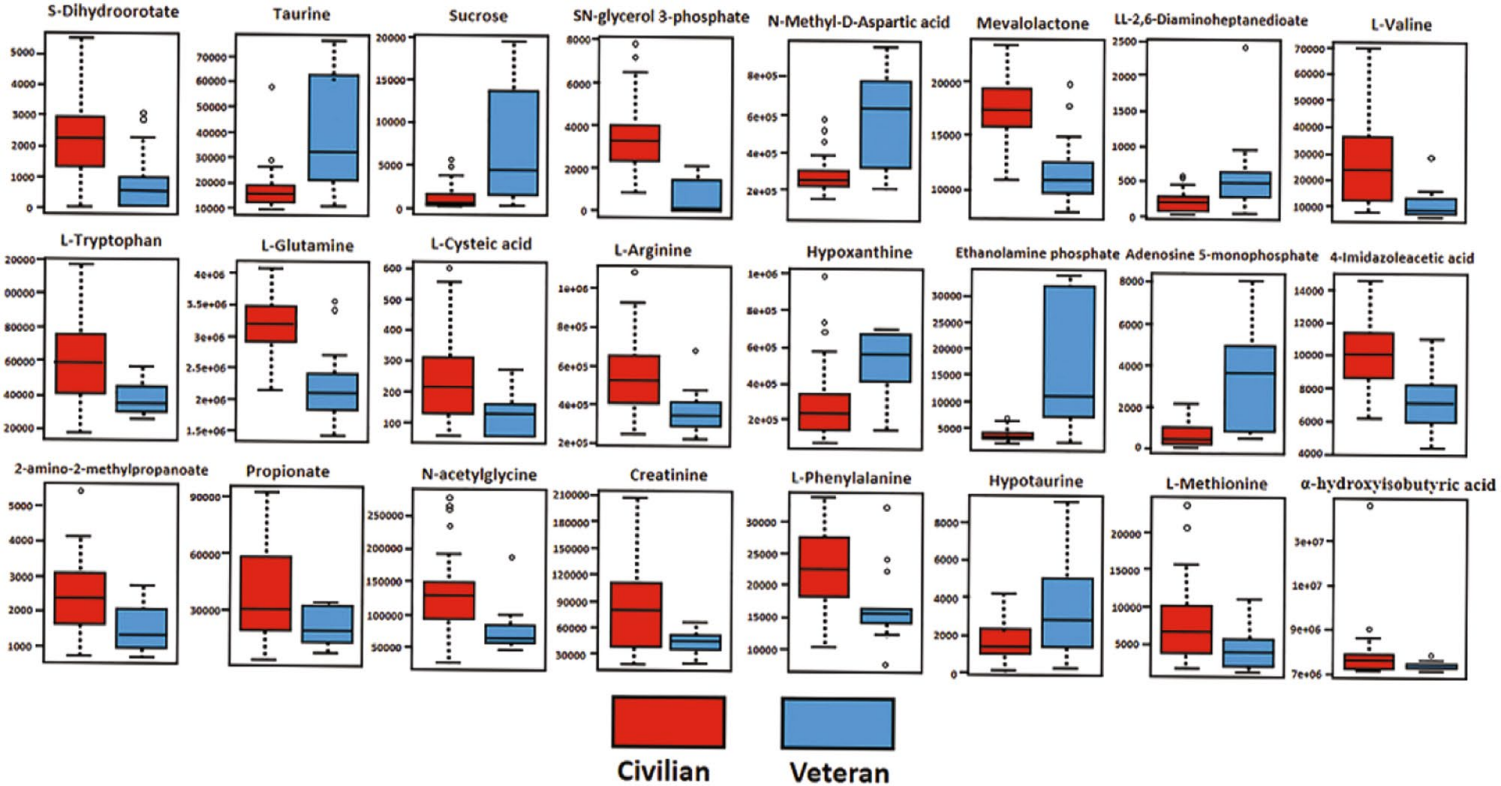
Shows 24 metabolites differentially expressed (p < 0.05), with significant FDR (p < 0.05) between the veteran and civilian sarcoidosis cohorts using the HILIC-MS dataset.

Unpaired T-tests on the sarcoidosis populations were performed using 55 and 103 metabolites identified by ^1^H-NMR and HILIC-MS, respectively. The T-test analysis on the ^1^H-NMR data showed 8 metabolites that were significantly (p < 0.05) different between the veteran and civilian cohorts with 6 having a significant low false discovery rates (FDR) (<0.05) (Table S1 & Fig. 2). T-tests on the HILIC-MS dataset showed 38 metabolites differentially expressed (p < 0.05), from which 24 metabolites had significant FDR (p < 0.05) (Table S2 & Fig. 3). These results confirm that the two sarcoidosis populations are metabolomically different.

### Veterans and civilians with sarcoidosis have different metabolomic profiles compared to veterans with COPD

MVA analysis also showed very distinct metabolic profiles for the sarcoidosis civilian cohort and the COPD cohort for both HILIC-MS (Q^2^ = 0.724) and ^1^H-NMR (Q^2^ = 0.713) (Figs. S9B and S10B). However, a smaller difference occurred between the two veteran cohorts. The predictability (Q^2^Y = 0.408 and 0.46) and significant difference (*p* = 0.00012 and 0.0001) for both ^1^H-NMR and HILIC-MS methods were considerably lower between the two veteran cohorts (Table 2) as shown in Figs. S9A and S10A. Also, a univariate approach using unpaired t-test proved that the metabolite alterations were more significant between civilian sarcoidosis and veterans with COPD than between the two veterans’ groups (Table S3–6).

### Metallomics study

#### PCA of the three study groups show significant clustering

In a targeted approach, 33 trace elements were quantified in the plasma samples of the three cohorts (civilians with sarcoidosis, veterans with sarcoidosis and veteran controls with COPD) using ICP-MS techniques. PCA analysis (Fig S11) shows a very clear separation of veteran patients with sarcoidosis from civilians with sarcoidosis. Similar to metabolomics results, this discrepancy was more noticeable between civilian sarcoidosis vs. COPD than veteran sarcoidosis vs. COPD (Figs. S12 and S13).

### Veteran sarcoidosis showed significant different metallomic profile compared to civilian sarcoidosis

**Figure 4:**
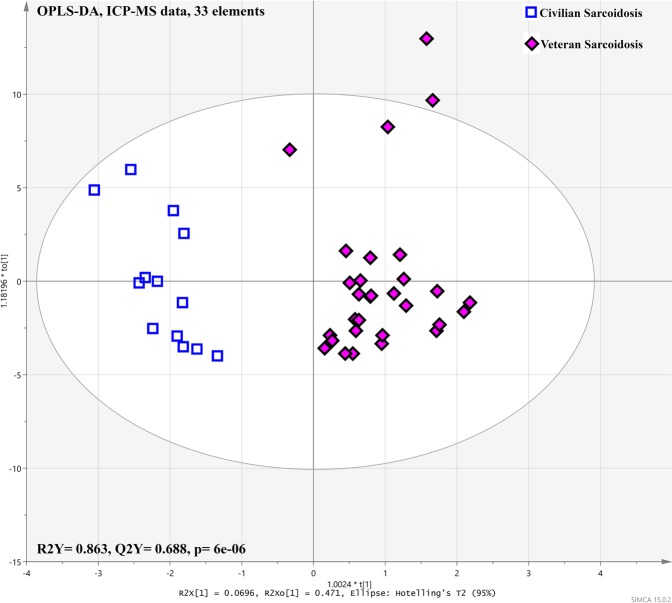
Shows 33 elements differentially detected (p < 0.05), with significant FDR (p < 0.05) between the veteran and civilian sarcoidosis cohorts using the ICP-MS dataset.

OPLS-DA analysis revealed a very good, highly predictive (Q^2^Y = 0.688) and significant (*p* = 6.003 × 10^−6^) model to discriminate between the two sarcoidosis groups based on the 33 elements (Fig. 4). The coefficient plot (Fig. S14) depicts differences in 33 elements between both cohorts showing increased rubidium (^85^Rb), gallium (^71^Ga), nickel (^60^Ni), cesium (^133^Cs), arsenic (^75^As), barium (^137^Ba), aluminum (^127^Al) and mercury (^202^Hg) and decreased boron (^11^Ba), antimony (^121^Sb), cadmium (^114^Cd) bromine (^79^Br), cobalt (^59^Co), Calcium (^44^Ca), selenium (^77^Se), strontium (^88^Sr), palladium (^105^Pd), magnesium (^24^Mg) and lead (^208^Pb) in the plasma samples of the veterans with sarcoidosis compared to the civilian cohort. Unpaired t-test, a univariate approach, showed 18 elements that were significantly different (p < 0.05) between the two sarcoidosis groups, including 10 elements with significant FDR (<0.05) (Table S7). It has been shown that other elements such as iron (^56^Fe) and manganese (^55^Mn) were significantly increased in the plasma of veteran sarcoidosis compared to civilian cohort. Permutation test confirmed the validation of R^2^ and Q^2^ values of each predictive model (OPLS-DA) using 200 repetitions.

### Metallomics profiles were significantly different between subjects with sarcoidosis and COPD

OPLS-DA analyses showed closer metallomics profiles between the two veteran groups compared to the civilian cohort. Utilizing the 33 elements, OPLS-DA analysis was highly predictive (Q^2^Y = 0.81) in separating civilian sarcoidosis subjects from veterans with COPD, while it was a less predictive discriminant model (Q^2^Y = 0.53) in separating veteran sarcoidosis subjects from veterans with COPD, using 29 elements (Fig. S15A,B). Additionally, individual t-tests provided more elements that were significantly (p < 0.05) changed when comparing civilian subjects with sarcoidosis versus veterans with COPD compared to veterans with sarcoidosis versus veterans with COPD (Tables S8 and S9). Overall, our metallomics results were remarkably similar to our metabolomics studies, likely reflecting different pathophysiological mechanisms of sarcoidosis in veterans compared to civilians.

### Correlation of metabolomic and metallomic profiles and sarcoidosis cohort status and clinical composite physiological index (CPI)

PLS-regression showed a strong relationship between predictive metabolomics profiles in distinguishing the veteran and civilian cohorts with sarcoidosis. R^2^ values were 0.95 and 0.91 for the HILIC-MS and the ^1^H-NMR metabolomic data, respectively (Fig. S16). The relation was weak between metabolomics profiles and CPI as an independent variable (R^2^ = 0.55 for HILIC-MS and R^2^ = 0.77 for ^1^H-NMR). R^2^ values for the regression were obtained using the PLSR method based on the most important metabolites obtained by the OPLS-DA analysis. Moreover, logistic regression showed that CPI is not a good predictive marker to discriminate between the two sarcoidosis cohorts (Table 1).

A similar PLS-regression analysis on metallomics profiles demonstrated a weaker correlation (R^2^ = 0.80) than metabolomics in discriminating between the sarcoidosis cohorts (Fig. S17). There was not a strong relationship between metallomics data in discriminating sarcoidosis from COPD.

### Relation of Metabolomics profiles and radiologic sarcoidosis stage

**Figure 5:**
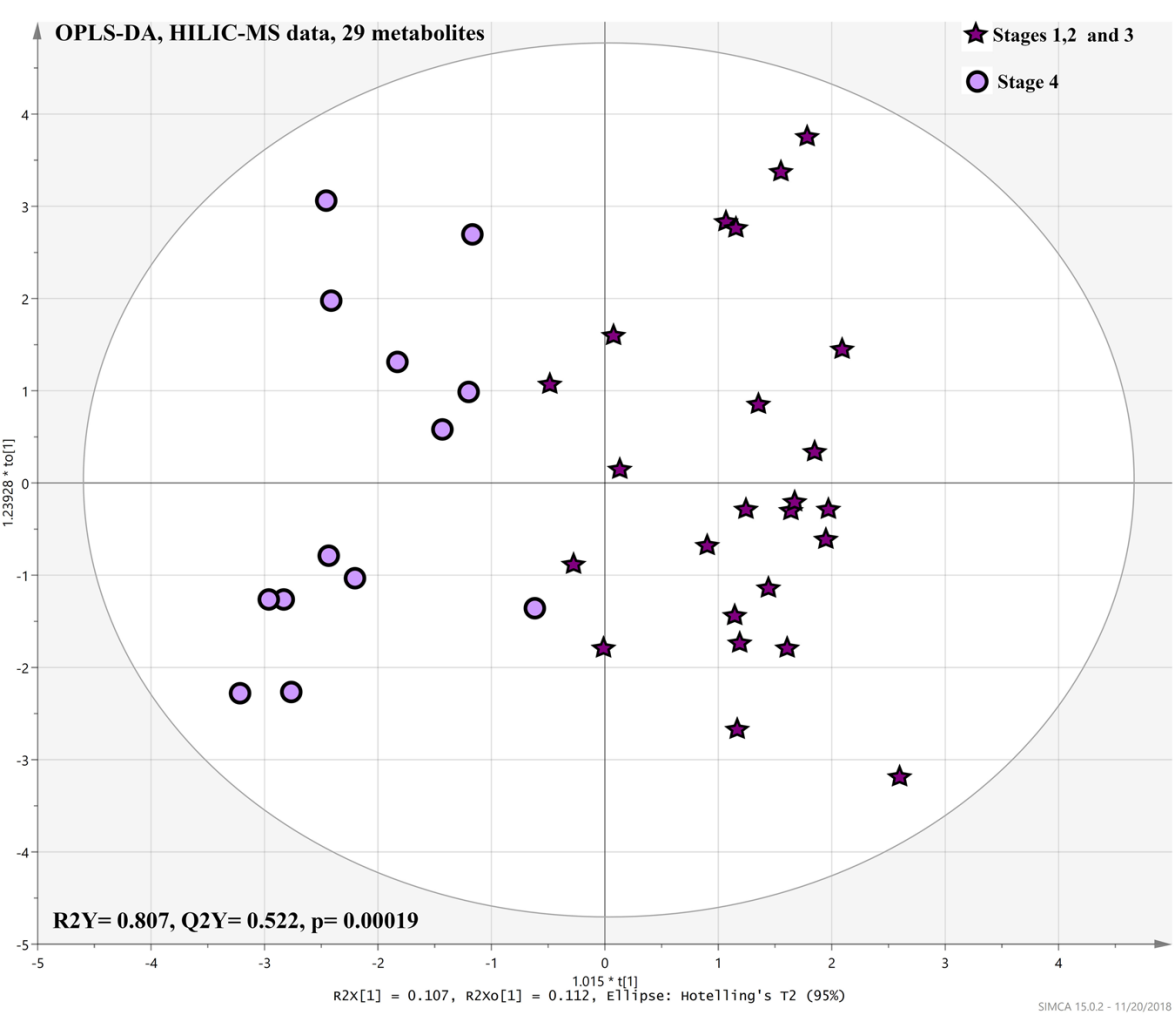
Shows OPLS-DA results from 29 metabolites that differentially expressed (p < 0.05), with significant FDR (p < 0.05) between the stage 4 and stages 1, 2, and 3 pulmonary sarcoidosis among both veteran or civilian population using the HILIC-MS dataset.

Subjects with stage 4 sarcoidosis different from other stages in their metabolomics profile (HILIC-MS data) with a high degree of predictability and a statistic significant difference (Q^2^Y = 0.522, *p* = 0.00019) (Fig. 5). Twenty-nine metabolites contributed in the differentiation of the radiologic sarcoidosis stages. Coefficient plots that illustrate the relative correlation of metabolites characteristic of sarcoidosis stage 4 vs. stages 1–3 is shown in Fig. S18. Univariate analysis showed only 7 metabolites that significantly (p < 0.05) differed between stages 1–3 and stage 4, which were already noted in the multivariate analysis (Table S10).

### Integration of ^1^H-NMR and HILIC-MS datasets

The ^1^H-NMR and HILIC-MS metabolomics datasets were further integrated using a normalized and block scaled method. Although there were several overlapped metabolites, both methods used different approaches for metabolite identification. The OPLS-DA model obtained, showed a slight improvement in separating the two sarcoidosis cohorts (Q^2^Y = 0.626) based on the 72 metabolites compared to the HILIC-MS dataset alone. Similar results were obtained for the discrimination between veterans with sarcoidosis and COPD, with a Q^2^Y = 0.693 and Q^2^Y = 0.843, respectively. Therefore, the combination of the two datasets further illustrates the metabolomic similarity of the two veteran cohorts when compared with the civilian sarcoidosis group.

### Pathway analysis

We applied parallel pathways analysis by MetaboAnalyst 3.0 and Cytoscape 3.6.0 using most differentiating metabolites obtained by the HILIC-MS and ^1^H-NMR platforms. Table 3 summarizes the details of the significant (p < 0.05) biochemical pathways with impact factor >1.0 after integration of both datasets. Using Cytoscape, the most important upregulated and downregulated biochemical pathways between sarcoidosis cohorts was graphically illustrated. We found that pathways involved in the transport of inorganic cations/anions and amino acids/oligopeptides, creatine metabolism, amino acid synthesis, and interconversion (transamination), amino acid transport across the plasma membrane, transport of glycerol from adipocytes to the liver by aquaporins, glycerophospholipid catabolism, and GABA biosynthesis, metabolism, and catabolism could be the most relevant biological networks that differ between veterans and civilians with sarcoidosis. A more detailed pathway analysis for both individual ^1^H-NMR and HILIC-MS datasets is shown in the supplementary section. (Tables S12 and S13).

**Table 3:** Pathway analysis based on selected differential metabolites after integration of both NMR and LC-MS metabolomics datasets.

Biochemical Pathway		Total	Expected	Hits	Raw p	FDR	Impact
1	Alanine, aspartate and glutamate metabolism	**24**	**0.57831**	**8**	**3.78e-08**	**1.01e-06**	**0.54606**
2	Taurine and hypotaurine metabolism	**20**	**0.48193**	**4**	**0.001103**	**0.012609**	**0.51439**
3	Arginine and proline metabolism	**77**	**1.8554**	**9**	**6.56e-05**	**0.001311**	**0.50307**
4	Glycine, serine and threonine metabolism	**48**	**1.1566**	**6**	**0.000843**	**0.011242**	**0.30843**
5	beta-Alanine metabolism	**28**	**0.6747**	**3**	**0.028381**	**0.13356**	**0.28022**
6	Glycerolipid metabolism	**32**	**0.77108**	**3**	**0.040164**	**0.17851**	**0.23544**
7	Histidine metabolism	**44**	**1.0602**	**4**	**0.020117**	**0.11495**	**0.19034**
8	Glycerophospholipid metabolism	**39**	**0.93976**	**4**	**0.013324**	**0.089586**	**0.17075**
9	Glyoxylate and dicarboxylate metabolism	**50**	**1.2048**	**2**	**0.34069**	**0.82592**	**0.14685**
10	Methane metabolism	**34**	**0.81928**	**3**	**0.046852**	**0.19727**	**0.14633**
11	D-Glutamine and D-glutamate metabolism	**11**	**0.26506**	**2**	**0.0273**	**0.13356**	**0.13904**
12	Phenylalanine metabolism	**45**	**1.0843**	**3**	**0.092443**	**0.32154**	**0.11906**
13	Tryptophan metabolism	**79**	**1.9036**	**1**	**0.85903**	**1**	**0.10853**
14	Nicotinate and nicotinamide metabolism	**44**	**1.0602**	**3**	**0.087726**	**0.319**	**0.10565**

FDR: False discovery rate.

## Discussion

This study shows that veterans with sarcoidosis have distinct metabolomic and metallomic profiles when compared to civilians with sarcoidosis. Since these populations have different environmental and occupational exposures, our findings agree with the notion that sarcoidosis is an abnormal inflammatory condition in response to many (still unidentified) triggers that may have their own particular pathophysiologic signature. Not surprisingly, the metabolic profiles among the two veteran cohorts studied (sarcoidosis and COPD) showed more similarity between them compared to civilians with sarcoidosis.

A number of reviews and research studies have addressed the impact of “exposomes” in altering metabolomics signatures and the role of metabolomics in characterizing particular environmental factors^28–31^, but to the best of our knowledge, no study has experimentally demonstrated this relation clearly when related to environmental exposure and sarcoidosis. The application of metabolomics in studying sarcoidosis has been reported previously. Lactate, acetate and N-butyrate is increased in saliva and 3-hydroxybutyrate, acetoacetate, carnitine, cystine, homocysteine, pyruvate, and trimethylamine N-oxide are some metabolites increased in the serum samples of patients with sarcoidosis compared to normal controls, while serum methanol, butyrate in saliva and isoleucine, glutamine and succinate are decreased^32,33^. We previously showed LC-MS-based metabolomics could differentiate fibrosing from non-fibrosing pulmonary sarcoidosis as well as differentiate subjects with composite phycological index (CPI) > 40 versus those that had CPI < 40^34^.

In this study we used metabolomics as a tool to characterize the exposome and investigate the molecular fingerprints and key cellular processes that are likely activated by exposure to the environment. The differences in metabolite alterations were readily detectable by both ^1^H-NMR and HILIC-MS detection methods, but most notably by the latter. Veterans with sarcoidosis have unique exposures and this may dictate the development of a particular type of sarcoidosis. For example, Gulf War Illness (GWI) is a syndrome described in deployed military to the Persian Gulf region exposed to particular environmental threats such as dust storms^35^. It is a multisystem disease diagnosed based on the Kansas criteria (presence of 3 or more of following chronic symptoms: fatigue, sleep problems, pain syndrome, neurologic and cognitive symptoms, gastrointestinal, respiratory and skin symptoms)^35^. Targeted metabolomics studies have shown GWI is a unique metabolic syndrome that is characterized with increased sphingolipids and phosphatidylcholines and decreased purines and endocannabinoids^36^, a specific metabolic fingerprint different to other conditions with similar symptomatology such as myalgic encephalomyelitis/chronic fatigue syndrome (ME/CFS)^36^. Furthermore, the lipid profile of GWI can be replicated in rat and mouse models exposed to a nerve gas antidote, pyridostigmine bromide^37,38^. On the other hand, mounting evidence has indicated that environmental exposure such airborne inorganic dust could be risk factors for sarcoidosis^39^ and that the nature of the exposure can impact the frequency and clinical phenotypes of the disease^40^. In mouse models, exposure to silica dust leads to metabolomic alterations after lung inflammation^41^. Similarly, we present here significant differences in metabolomic profiles in 2 sarcoidosis populations with different exposures.

One of the significant metabolomic changes observed was in amino acids. Compared to civilians, veterans with sarcoidosis exhibited lower concentration in most amino acids analyzed molecules important in the induction of anti-inflammatory mechanisms and reducing stress response in inflammation^42,43^. Amino acids such as glutamine, phenylalanine, tryptophan, arginine and cysteine play a vital role in immunomodulation, particularly through the T-cell proliferation and activation^44^. Veterans also exhibited an increased concentration in some anti-inflammatory biomarkers such as taurine, hypoxanthine, glucosamine, reflecting a high degree of interactions between both anti-inflammatory and pro-inflammation mechanisms.

Exposure to metallic environmental factors, including occupational and infectious causes, have been associated with sarcoidosis^45,46^. For example, exposure to beryllium, aluminum, rare earth elements, and titanium have been associated with sarcoidosis in mine, manufacturer, and agriculture workers^46–49^. We found significantly higher in the plasma concentration of rubidium, gallium, nickel, and cesium in the plasma of veterans compared to civilians with sarcoidosis. Veterans deployed to the Middle East have particular exposures to heavy metal and trace elements. For example, Pb, Zn, Cd, Ni, and Cr contamination of seawater, food, and soils due to the Gulf War and oil spills have been reported^50,51^. Military operations by themselves have added another potential source of contamination of water and soil in the gulf, During the two Gulf Wars in the 1990s, millions of hectares were contaminated due to spilling of millions of oil barrels and burning of hundreds of oil wells and is estimated that 340 tons of uranium was depleted in the first Gulf War^52^. Accordingly, we have shown that the plasma level of uranium in veterans with sarcoidosis is higher than was observed in the civilian cohort. Furthermore, nickel contamination from different sources such as batteries can have effect on lung and antioxidant system in the form of water-soluble nickel phosphate^53^. Nickel was also higher in the veteran sarcoidosis group.

The lung is the first line of exposure that is exposed to essential or non-essential/toxic elements and potentially toxic metal-related nanomaterials. Nanoparticles generated from dust storms or gun powders may deposit in the lungs and direct activated alveolar macrophages toward the pulmonary interstitium causing a more severe immune response than larger particles^54^. Metalloids become localized and interact with pulmonary cells and tissues or are dispersed all over the body by passing biological barriers^55^. Metal ions have significant biological effects in different metabolic pathways^56,57^. For example, radioactive Cesium 137 (^137^Cs) has been associated with reduced lung function in children in the Chernobyl disaster^58^.

Dust storms in deployment areas represent an additional exposure hazard due to their high contents of silica, aluminum, and rare earth elements^59,60^. The association of silica exposure and development of sarcoidosis was studied by Rafnsson *et al*.^61^, who found that people who were exposed to dust had up to a 5 times higher risk of development of pulmonary sarcoidosis when compared to a normal population (9.3 vs. 0.5–2.7 in 100,000, respectively). Dust storms also contain particles such as, calcium oxide (CaO) and magnesium oxide (MgO), and oxides of sodium and potassium (Na^2^O and K^2^O) as well as silicon dioxide (SiO^2^), aluminum oxide (Al^2^O^3^), iron (Fe^2^O^3^) and titanium (TiO^2^) oxides and trace elements such as, zirconium (Zr), strontium (Sr), rubidium (Rb)^62^. Our data showed that Mg, Ca, Al, Ti, and Fe is increased in veterans with sarcoidosis compared to the civilian sarcoidosis population. Investigations on dust in the Iraq desert revealed that the quartz particle is surrounded by calcium carbonate containing various elements such as aluminum, iron, uranium, nickel, cobalt, copper, lead, chromium, strontium, tin, manganese, zinc, barium, arsenic, and vanadium^63^. Accordingly, we observed an increase in most of the aforementioned elements in the plasma samples of veteran sarcoidosis. Of note, cadmium, known to be involved in the pathogenesis of pulmonary carcinogenesis^64^, was not higher among the veterans studied.

We also noted that veterans with sarcoidosis have higher concentrations of elements that regulate biologic processes in immune cells. For instance, lanthanum (a rare earth element) can compete with calcium in different proteins^65,66^. This substitution can alter the function of annexin A11 (ANXA11), an intracellular metalloprotein that carries 5 calcium ions. It has been proposed that alteration of ANXA11 is associated with pulmonary fibrosis in sarcoidosis^67^. In this way, sarcoidosis in veterans may be considered an occupational disease.

Another interesting finding noted in veterans with sarcoidosis was with pathway analysis, which showed that GABA synthesis and metabolism might be downregulated in veterans compared to civilian with sarcoidosis, with both precursor metabolites of GABA (glutamate or glutamine) and breakdown metabolites of GABA (succinate semialdehyde) were decrease in veterans. GABA can modulate the immune response via secretion of cytokines, activation, proliferation and migration of immune cells^68^.

The current study has two major limitations. A relatively low sample size in our study may lead to a lack of precision and the results have not been validated in an external validation cohort. To partially overcome the limitations, we applied 3 different methods to validate the findings with higher resolution. The very similar results obtained using three different techniques, ^1^H-NMR, HILIC-MS and ICP-MS, serves as an important internal validation of our results.

Using metabolomics and metallomics, this study provides relevant evidence for the role of potential environmental risk factors for different molecular phenotypic responses of sarcoidosis. More investigation will be needed to identify biochemical pathways that link between environmental risk factors and a disease phenotype as well as metal-related metabolomics perturbations in human diseases. We conclude then that this comprehensive metabolite and metallomic profiling clearly shows distinct metabolomic profiles between two sarcoidosis populations exposed to different environmental factors. Metabolic fingerprints can be a powerful and useful tool for sarcoidosis phenotyping in a more accurate manner than the current clinical approach. Furthermore, characterization of metabolic profiles is a promising platform to better understand the underlying pathophysiological mechanisms, establish the importance of environmental exposures and determine disease severity, prognosis, and response to treatment.

## Supplementary information

Supplementary Info
