# Supplementary material for: Metabolomic and metallomic profile differences between Veterans and Civilians with Pulmonary Sarcoidosis
Source: Sci Rep. 2019 Dec 20;9:19584. doi: 10.1038/s41598-019-56174-8 (PMC6925242; doi:10.1038/s41598-019-56174-8)
Supplement: Supplementary file 1 — Supplementary Info [file 41598_2019_56174_MOESM1_ESM.pdf]

Metabolomic and metallomic profile differences between Veterans and Civilians with Pulmonary Sarcoidosis

Mohammad Mehdi Banoei, Isabella Iupe, Reza Dowlatabadi Bazaz, Michael Campos, Hans J Vogel, Brent W. Winston, Mehdi Mirsaeidi

Supplementary

^{1}H NMR Spectroscopy

Sample Preparation

200 µl of samples were thawed on the ICE and were them filtered using 3 kDa NanoSep microcentrifuge filters at 12,000 x g for 1 hour at 4ºC. Filters were prewashed before adding samples using ddH_2O to reduce preservative contamination. After collecting samples into clean 1.5 ml vials, filters were additionally rinsed using 100 µl of D_2O. 80 µl NMR phosphate buffer was added to samples and sample were adjusted using D_2O to final volume 400 µl. NMR phosphate buffer consisted of 0.5 M NaH_2PO_4 buffer solution at pH 7.0) containing 2.5 mM 2,2-dimethylsilapentane-5-sulfonate (DSS, final concentration 0.5 mM) as an internal reference compound. To prevent bacterial contamination, 10 µl sodium azide (1 M NaN_3) was added to each sample. pH was adjusted to 7.0 ± 0.04 at room temperature.

Data Acquisition

NMR spectroscopy was carried out in a blinded manner using an automated sample changer hold at 4ºC on a a 600 MHz Bruker Ultrashield Plus NMR spectrometer (Bruker BioSPin Ltd., Canada). NMR spectra were acquired by pre-saturation pulse sequence (noesypr 1d) with an optimized water suppression and a mixing time of 100 ms [1, 2]. NMR spectra were obtained by 1024 scan, zero filled and Fourier transformed to 128k points. NMR spectra were processed by Topspin software program to line broadening, phasing, baseline correction and referencing peaks compared to the DSS peak at 0.0 ppm using the (Bruker BioSpin Ltd., Canada).

Metabolite Concentration Profiling

NMR spectra were randomly ordered and analyzed to avoid progressive bias. ChenomX NMR Suite 7.1 software (Chenomx Inc., Edmonton, Alberta, Canada) was used to identify and quantify metabolites of NMR spectra [1]. NMR spectra was manually phased in the processor module of the ChenomX software. Water region and baseline correction was deleted to ease profiling of the peaks. All peaks were automatically quantified compare to the DSS concentration, as an internal reference [3].

Inductively Coupled Plasma Mass Spectrometry (ICP-MS) analysis

Sample Preparation

Plasma samples were prepared using OmniTrace® nitric acid (EMD Millipore Inc., Darmstadt, Germany) diluted (2%) in high purity water which obtained by a Milli-Q® Gradient A10® water purification system (Millipore S.A.S, Molsheim, France) with a resistivity of 18.2. Fifty µl of plasma samples were centrifuged at 13200 rpm for 10 minutes at 4°C temperature. Supernatant was then transferred to a 15 ml tubes containing 10 ml of 2% nitric acid to obtain a 1:200 dilution. Diluted samples in acid nitric were centrifuged at 4000 rpm for 10 minutes to make the samples as clean as possible without debris. SeronormTM trace elements (SERO AS, Billingstad, Norway) serum level 2 (lot 1309416) was used during experiment of samples as the quality controls (QCs) to evaluate the reproducibility and lowering of sensitivity due to contamination of the device. The preparation of Seronorm was done based on the manufacturers' instruction by dissolving in water followed by dilution in 2% nitric acid.

Data analysis

Both NMR and HILIC-MS datasets were normalized by median fold change normalization data [9]. Log transformed and centering univariance scaling (UV) were applied to preprocessed datasets. Multivariate data analysis wes performed then analyzed using the SIMCA-P+ program (Version 13.0, Umetrics AB, Umeå, Sweden).

Verification of the OPLS-DA Model

CV-ANOVA, R_2Y and Q_2Y were considered to verify the quality of OPLS-DA, a supervised multivariate separation analysis. CV-ANOVA determine the reliability and assess the significance of discrimination model. R_2Y and Q_2Y explain the goodness of variation of group status and the goodness of prediction, respectively [10]. Both R_2Y and Q_2Y is obtained through a cross validation method which was based on the internal sevenfold cross validation by leaving out of one seventh ($1/7_{th}$) to test R_2Y on remaining portion and measuring the prediction (Q_2Y) using $1/7_{th}$ removed. This process is repeated 7 times for each for each left out group of samples. R_2Y and Q_2Y score can vary between 0 and 1, where the scores are closer to 1 showing an excellent with a high level of predictability. Q_2Y more than 0.5 is considered a good model and high predictability for human samples. $Q_2Y > 0.7$ is a very good model.

Variables important on projection (VIP)

VIP was used to choose the best OPLS-DA model regarding the highest predictability. The VIP is used to select the metabolites with the highest effects in separation of the two groups. VIP

works in a weighted fashion using a quantitative measure of discriminatory power of the metabolites that is ranked by a unitless number.

Supplementary (Figures and Tables)

	Name	Mean (SD) of Civilian	Mean (SD) of Veteran	p-value	q-value (FDR)	Fold Change	Civilian/Veteran
1	**Glutamate**	0.626 (0.199)	0.876 (0.258)	0.0013	0.022	-1.4	Down
2	**Glutamine**	3.428 (0.552)	2.908 (0.488)	0.0054	0.0499	1.18	Up
3	**Valine**	1.341 (0.340)	1.129 (0.196)	0.0143	0.0987	1.19	Up
4	**Phenylalanine**	0.401 (0.071)	0.343 (0.074)	0.0192	0.1172	1.17	Up
5	**Acetone**	0.062 (0.055)	0.034 (0.005)	0.0006 (W)	0.022	1.83	Up
6	**Lactate**	6.755 (4.129)	8.985 (3.311)	0.0008 (W)	0.022	-1.33	Down
7	**Pyruvate**	0.362 (0.130)	0.506 (0.142)	0.0016 (W)	0.022	-1.4	Down
8	**O-Phosphocholine**	1.469 (0.718)	1.903 (0.380)	0.0032 (W)	0.0354	-1.3	Down

Table S1, Unpaired t-test analysis of the $_1$H-NMR dataset showing significant ($p < 0.05$) metabolites between the veteran and civilian sarcoidosis cohorts. (Highlight are metabolites with FDR < 0.05)

	Name	Mean (SD) of Civil	Mean (SD) of Veteran	p-value	q-value (FDR)	Fold Change	Civil/Veteran
1	**L-TRYPTOPHAN**	59818.516 (22822.083)	38286.558 (10311.448)	0.0001	0.0024	1.56	Up
2	**4-IMIDAZOLEACETIC ACID**	10274.649 (2032.768)	7434.076 (2100.625)	0.0002	0.0026	1.38	Up
3	**CREATININE**	129809.818 (74087.331)	73598.561 (22974.247)	0.0006	0.0051	1.76	Up
4	**PROPIONATE**	64919.259 (38865.519)	36747.736 (15794.559)	0.0016	0.0107	1.77	Up
5	**L-ARGININE**	547222.030 (191290.579)	365799.915 (125084.457)	0.0032	0.0189	1.5	Up
6	**2-AMINO-2-METHYLPROPANOATE**	2429.040 (1022.633)	1548.279 (688.889)	0.0072	0.0348	1.57	Up
7	**L-PHENYLALANINE**	22650.486 (6479.732)	16856.945 (6293.595)	0.0096	0.0402	1.34	Up
8	**L-TYROSINE**	163744.541 (60274.494)	116479.185 (38754.084)	0.0131	0.0503	1.41	Up
9	**D---ARABINOSE**	272529.363 (106243.233)	214717.515 (40577.038)	0.0137	0.0506	1.27	Up
10	**HYPOTAURINE**	1681.570 (862.075)	3468.456 (2611.048)	0.0312	0.0919	-2.06	Down
11	**PYRUVIC ALDEHYDE**	1566110.773 (572784.595)	1279341.677 (275797.991)	0.0326	0.0931	1.22	Up
12	**MEVALOLACTONE**	17493.580 (2883.486)	12040.933 (3522.546)	< 0.0001	0	1.45	Up
13	**L-GLUTAMINE**	3189427.400 (492534.572)	2237994.077 (660559.642)	< 0.0001	0.0003	1.43	Up
14	**SN-GLYCEROL 3-PHOSPHATE**	3527.694 (1723.584)	720.341 (826.685)	< 0.0001 (W)	0	4.9	Up
15	**ETHANOLAMINE PHOSPHATE**	3923.612 (1273.355)	16890.127 (12760.581)	< 0.0001 (W)	0.0008	-4.3	Down
16	**ADENOSINE 5-MONOPHOSPHATE**	662.188 (610.429)	3408.038 (2487.978)	0.0002 (W)	0.0026	-5.15	Down
17	**L-VALINE**	51210.494 (32268.635)	22592.352 (12503.570)	0.0003 (W)	0.0036	2.27	Up
18	**SUCROSE**	1245.714 (1640.351)	7128.660 (6806.609)	0.0004 (W)	0.0037	-5.72	Down
19	**N-METHYL-D-ASPARTIC ACID**	282501.203 (102414.996)	567440.446 (259325.433)	0.0004 (W)	0.0037	-2.01	Down
20	**LL-2-6-DIAMINOHEPTANEDIOATE**	227.766 (160.646)	626.225 (592.339)	0.0013 (W)	0.0102	-2.75	Down
21	**HYPOXANTHINE**	302738.254 (215465.157)	519286.500 (177904.605)	0.0013 (W)	0.0102	-1.72	Down
22	**TAURINE**	877789.610 (456386.257)	1882545.177 (1156215.298)	0.0014 (W)	0.0103	-2.14	Down
23	**DL-5-HYDROXYLYSINE**	1820.929 (1728.239)	916.723 (833.259)	0.0029 (W)	0.0182	1.99	Up
24	**S-DIHYDROOROTATE**	2364.075 (1471.159)	945.036 (1116.190)	0.0034 (W)	0.0189	2.5	Up
25	**L-HISTIDINE**	550310.557 (155922.474)	432563.400 (107080.389)	0.0061 (W)	0.0323	1.27	Up
26	**N-ACETYLGLYCINE**	128390.289 (69850.471)	78676.424 (37731.655)	0.0073 (W)	0.0348	1.63	Up
27	**SUCCINATE SEMIALDEHYDE**	219845.997 (278290.059)	101654.540 (38346.462)	0.0080 (W)	0.0362	2.16	Up
28	**MANNOSE**	12023482.967 (4155821.609)	9570963.615 (2075416.765)	0.0088 (W)	0.0384	1.26	Up
29	**L-CYSTEIC ACID**	239.262 (153.036)	115.854 (66.856)	0.0119 (W)	0.0476	2.07	Up
30	**NALPHA-ACETYL-L-LYSINE**	5061.757 (1421.164)	4009.379 (731.646)	0.0142 (W)	0.0506	1.26	Up
31	**D-GULONIC ACID GAMA-LACTONE**	3488.693 (3409.347)	2358.048 (4337.089)	0.0208 (W)	0.0717	1.48	Up
32	**L-ASPARAGINE**	122904.117 (28480.160)	105323.019 (27567.226)	0.0241 (W)	0.0803	1.17	Up
33	**N-ACETYL-L-ASPARTIC ACID**	20759.889 (12973.888)	13819.238 (5355.247)	0.0278 (W)	0.0897	1.5	Up
34	**L-METHIONINE**	25656.645 (10977.821)	18724.125 (5671.912)	0.0298 (W)	0.0905	1.37	Up
35	**ALPHA-HYDROXYISOBUTYRIC ACID**	2296896.053 (3364682.580)	1030699.000 (495582.266)	0.0298 (W)	0.0905	2.23	Up
36	**L-SERINE**	216076.357 (60414.378)	175777.815 (41413.084)	0.0367 (W)	0.102	1.23	Up
37	**GLYCOLALDEHYDE DIMER**	88187.885 (163373.366)	29679.243 (25621.056)	0.0420 (W)	0.1135	2.97	Up
38	**SUBERIC ACID**	36226.638 (29389.895)	20756.387 (7616.248)	0.0449 (W)	0.1181	1.75	Up

Table S2. Unpaired t-test analysis of HILIC-MS dataset showing significant ($p < 0.05$) metabolites between the veteran and civilian sarcoidosis cohorts. (Highlight shows metabolites with FDR < 0.05)

	Name	Mean (SD) of COPD	Mean (SD) of civilian Sarcoidosis	p-value	q-value (FDR)	Fold Change	Sarcoidosis/COPD
1	**Phenylalanine**	0.020 (0.004)	0.024 (0.004)	0.0004	0.0026	-1.18	Up
2	**Dimethylamine**	0.004 (0.001)	0.005 (0.001)	0.055	0.1164	-1.16	Up
3	**2-Oxoisocaproate**	0.006 (0.003)	0.011 (0.004)	< 0.0001	0	-1.79	Up
4	**2-Oxoglutarate**	0.012 (0.006)	0.032 (0.017)	< 0.0001 (W)	0	-2.75	Up
5	**Isoleucine**	0.023 (0.007)	0.036 (0.013)	< 0.0001 (W)	0	-1.58	Up
6	**Isopropanol**	0.022 (0.016)	0.010 (0.028)	< 0.0001 (W)	0	2.17	Down
7	**Valine**	0.078 (0.019)	0.113 (0.029)	< 0.0001 (W)	0	-1.45	Up
8	**Taurine**	0.097 (0.041)	0.159 (0.079)	0.0001 (W)	0.0011	-1.63	Up
9	**Leucine**	0.044 (0.019)	0.060 (0.020)	0.0002 (W)	0.0014	-1.37	Up
10	**Methionine**	0.015 (0.012)	0.033 (0.021)	0.0003 (W)	0.0023	-2.24	Up
11	**Creatine**	0.018 (0.008)	0.025 (0.009)	0.0005 (W)	0.0026	-1.41	Up
12	**Tyrosine**	0.029 (0.007)	0.037 (0.012)	0.0008 (W)	0.0038	-1.31	Up
13	**Adipate**	0.003 (0.001)	0.005 (0.002)	0.0009 (W)	0.0044	-1.59	Up
14	**4-Hydroxybutyrate**	0.007 (0.003)	0.017 (0.016)	0.0010 (W)	0.0044	-2.29	Up
15	**Acetone**	0.012 (0.005)	0.011 (0.010)	0.0016 (W)	0.0064	1.14	Down
16	**Serine**	0.057 (0.051)	0.069 (0.027)	0.0041 (W)	0.0151	-1.22	Up
17	**Acetate**	0.022 (0.044)	0.043 (0.125)	0.0064 (W)	0.0222	-1.95	Up
18	**Beta-Alanine**	0.093 (0.061)	0.136 (0.075)	0.0077 (W)	0.0249	-1.46	Up
19	**Isobutyrate**	0.004 (0.003)	0.007 (0.010)	0.0093 (W)	0.0285	-1.82	Up
20	**Formate**	0.014 (0.006)	0.021 (0.017)	0.0099 (W)	0.0287	-1.46	Up
21	**Lactate**	0.876 (0.323)	0.736 (0.448)	0.0111 (W)	0.0288	1.19	Down
22	**Glycerol**	0.197 (0.051)	0.228 (0.070)	0.0113 (W)	0.0288	-1.16	Up
23	**Choline**	0.011 (0.008)	0.015 (0.011)	0.0115 (W)	0.0288	-1.47	Up
24	**Propylene glycol**	0.023 (0.053)	0.010 (0.014)	0.0146 (W)	0.035	2.19	Down
25	**Pyruvate**	0.047 (0.014)	0.040 (0.014)	0.0382 (W)	0.0854	1.17	Down
26	**Asparagine**	0.031 (0.015)	0.041 (0.020)	0.0388 (W)	0.0854	-1.3	Up

Table S3. Unpaired t-test analysis of NMR dataset showing significant ($p < 0.05$) metabolites between civilian sarcoidosis cohort and COPD. (Highlight shows metabolites with FDR < 0.05).

	Name	Mean (SD) of COPD	Mean (SD) of Veteran Sarcoidosis	p-value	q-value (FDR)	Fold Change	COPD/Sarcoidosis 1
1	**2-Oxoisocaproate**	0.006 (0.003)	0.009 (0.004)	0.0028	0.0174	-1.5	Up
2	**Pyruvate**	0.047 (0.014)	0.057 (0.016)	0.0418	0.1277	-1.21	Up
3	**4-Hydroxybutyrate**	0.007 (0.003)	0.023 (0.008)	< 0.0001 (W)	0	-3.13	Up
4	**Acetone**	0.012 (0.005)	0.006 (0.001)	< 0.0001 (W)	0	2.06	Down
5	**Methionine**	0.015 (0.012)	0.046 (0.008)	< 0.0001 (W)	0	-3.15	Up
6	**Taurine**	0.097 (0.041)	0.188 (0.080)	< 0.0001 (W)	0.0011	-1.94	Up
7	**Isopropanol**	0.022 (0.016)	0.008 (0.009)	0.0002 (W)	0.0018	2.89	Down
8	**2-Oxoglutarate**	0.012 (0.006)	0.025 (0.010)	0.0005 (W)	0.005	-2.09	Up
9	**Beta-Alanine**	0.093 (0.061)	0.156 (0.061)	0.0011 (W)	0.0084	-1.67	Up
10	**Valine**	0.078 (0.019)	0.096 (0.017)	0.0022 (W)	0.0151	-1.24	Up
11	**O-Phosphocholine**	0.074 (0.033)	0.103 (0.021)	0.0048 (W)	0.0262	-1.4	Up
12	**Isoleucine**	0.023 (0.007)	0.030 (0.009)	0.0054 (W)	0.0268	-1.3	Up
13	**Formate**	0.014 (0.006)	0.020 (0.008)	0.0097 (W)	0.041	-1.38	Up
14	**Leucine**	0.044 (0.019)	0.051 (0.011)	0.0097 (W)	0.041	-1.17	Up
15	**3-Hydroxyisovalerate**	0.002 (0.002)	0.007 (0.010)	0.0142 (W)	0.0559	-3.34	Up
16	**Propylene glycol**	0.023 (0.053)	0.015 (0.033)	0.0168 (W)	0.0618	1.53	Down
17	**Creatinine**	0.034 (0.009)	0.040 (0.007)	0.0191 (W)	0.0657	-1.18	Up
18	**Choline**	0.011 (0.008)	0.015 (0.006)	0.0357 (W)	0.1156	-1.39	Up
19	**3-Hydroxybutyrate**	0.045 (0.068)	0.016 (0.010)	0.0486 (W)	0.1407	2.87	Down

Table S4. Unpaired t-test analysis of NMR dataset showing significant ($p < 0.05$) metabolites between veteran sarcoidosis cohort and COPD. (Highlight shows metabolites with FDR < 0.05).

	Name	Mean (SD) of Civilian sarcoidosis	Mean (SD) of COPD	p-value	q-value (FDR)	Fold Change	Civil/COPD
1	MANNOSE	12023482.967 (4155821.609)	8266237.706 (2705069.100)	0.0001	0.0003	1.45	Up
2	D--ARABINOSE	272529.363 (106243.233)	183944.194 (70601.357)	0.0003	0.0009	1.48	Up
3	PYRUVIC ALDEHYDE	1566110.773 (572784.595)	1143224.288 (362822.988)	0.0011	0.0028	1.37	Up
4	METHYL VANILLATE	18958.969 (7541.545)	14748.321 (4700.940)	0.0112	0.0195	1.29	Up
5	MEVALOLACTONE	17493.580 (2883.486)	12966.064 (3256.238)	< 0.0001	0	1.35	Up
6	L-PHENYLALANINE	22650.486 (6479.732)	14791.734 (3537.189)	< 0.0001	0	1.53	Up
7	L-GLUTAMINE	3189427.400 (492534.572)	2372657.941 (548954.007)	< 0.0001	0	1.34	Up
8	L-ASPARAGINE	122904.117 (28480.160)	96898.444 (21232.708)	< 0.0001	0.0003	1.27	Up
9	L-ARGININE	547222.030 (191290.579)	347690.606 (116583.133)	< 0.0001	0	1.57	Up
10	DEOXYCHOLATE	322903.139 (141622.829)	187962.614 (104105.216)	< 0.0001	0.0002	1.72	Up
11	CREATININE	129809.818 (74087.331)	58374.791 (22550.003)	< 0.0001	0	2.22	Up
12	4-IMIDAZOLEACETIC ACID	10274.649 (2032.768)	8050.951 (1740.665)	< 0.0001	0	1.28	Up
13	4-HYDROXYBENZALDEHYDE	52660.861 (10682.123)	40593.043 (10922.843)	< 0.0001	0.0002	1.3	Up
14	3-ALPHA-11-BETA-17-ALPHA-21-TETRAHYDROXY- 5-ALPHA-PREGNAN-20-ONE	4263.420 (2502.254)	1825.514 (1191.736)	< 0.0001	0.0001	2.34	Up
15	S-DIHYDROOROTATE	2362.501 (1473.694)	771.794 (1028.553)	< 0.0001 (W)	0	3.06	Up
16	SN-GLYCEROL 3-PHOSPHATE	3527.694 (1723.584)	1012.787 (1013.139)	< 0.0001 (W)	0	3.48	Up
17	SARCOSINE	117744.636 (51809.512)	69167.648 (51761.680)	< 0.0001 (W)	0	1.7	Up
18	RESORCINOL MONOACETATE	57895.617 (92505.127)	16382.150 (64033.151)	< 0.0001 (W)	0	3.53	Up
19	PROPIONATE	64919.259 (38865.519)	27543.556 (20756.470)	< 0.0001 (W)	0	2.36	Up
20	NALPHA-ACETYL-L-LYSINE	5061.757 (1421.164)	3574.632 (792.437)	< 0.0001 (W)	0	1.42	Up
21	N-METHYL-L-GLUTAMATE	7799.845 (4408.937)	4207.634 (2558.014)	< 0.0001 (W)	0.0003	1.85	Up
22	N-FORMYLGLYCINE	43525.777 (43786.197)	18921.098 (7001.746)	< 0.0001 (W)	0	2.3	Up
23	N-ALPHA-ACETYL-L-ASPARAGINE	7679.840 (4070.181)	4455.756 (2078.995)	< 0.0001 (W)	0.0002	1.72	Up
24	LL-2-6-DIAMINOHEPTANEDIOATE	225.516 (163.163)	1244.641 (1765.986)	< 0.0001 (W)	0	-5.52	Down
25	L-VALINE	51210.494 (32268.635)	19209.066 (17252.406)	< 0.0001 (W)	0	2.67	Up
26	L-TYROSINE	163744.541 (60274.494)	100160.891 (29176.349)	< 0.0001 (W)	0	1.63	Up
27	L-METHIONINE	25656.645 (10977.821)	16612.641 (10795.283)	< 0.0001 (W)	0	1.54	Up
28	L-HISTIDINE	550310.557 (155922.474)	418923.971 (85965.980)	< 0.0001 (W)	0.0001	1.31	Up
29	DL-5-HYDROXYLYSINE	1820.929 (1728.239)	750.192 (624.087)	< 0.0001 (W)	0	2.43	Up
30	CREATINE	73375.340 (33510.168)	40920.300 (31912.284)	< 0.0001 (W)	0	1.79	Up
31	CHOLATE	56232.541 (24649.487)	28010.294 (16041.232)	< 0.0001 (W)	0	2.01	Up
32	4-METHYL-2-OXO-PENTANOIC ACID	1260994.433 (578940.279)	691326.344 (244980.000)	< 0.0001 (W)	0	1.82	Up
33	O-ACETYL-L-SERINE	48617.316 (31572.699)	29230.610 (12341.191)	0.0001 (W)	0.0004	1.66	Up
34	L-TRYPTOPHAN	59818.516 (22822.083)	39544.021 (11928.589)	0.0001 (W)	0.0003	1.51	Up
35	L-THREONINE	320588.157 (109063.811)	229896.053 (70962.737)	0.0001 (W)	0.0003	1.39	Up
36	L-SERINE	216076.357 (60414.378)	164008.271 (38448.103)	0.0001 (W)	0.0003	1.32	Up
37	ETHANOLAMINE PHOSPHATE	3923.612 (1273.355)	10988.774 (9561.906)	0.0001 (W)	0.0004	-2.8	Down
38	L-CYSTEIC ACID	237.998 (154.628)	118.704 (104.422)	0.0002 (W)	0.0007	2	Up
39	ADENOSINE 5-MONOPHOSPHATE	660.840 (611.821)	2375.488 (2142.537)	0.0005 (W)	0.0013	-3.59	Down
40	6-DEOXY-L-GALACTOSE	55383.869 (27182.563)	33216.185 (14286.962)	0.0005 (W)	0.0013	1.67	Up
41	N-ACETYL-L-ASPARTIC ACID	20759.889 (12973.888)	13826.156 (4228.769)	0.0015 (W)	0.0038	1.5	Up
42	TRANS-4-HYDROXYPROLINE	39508.363 (16993.694)	28751.865 (14461.930)	0.0016 (W)	0.0039	1.37	Up
43	ACETOIN	14296.614 (8340.230)	8374.084 (4233.445)	0.0020 (W)	0.0046	1.71	Up
44	SUCCINATE SEMIALDEHYDE	219845.997 (278290.059)	121712.168 (111029.569)	0.0023 (W)	0.0052	1.81	Up
45	D-GULONIC ACID GAMA-LACTONE	3488.693 (3409.347)	1958.569 (3025.617)	0.0025 (W)	0.0056	1.78	Up
46	TAURINE	877789.610 (456386.257)	1515848.274 (1041245.380)	0.0049 (W)	0.0106	-1.73	Down
47	PALMITATE	3403.990 (2287.601)	4691.507 (1767.054)	0.0049 (W)	0.0106	-1.38	Down
48	URATE	27870000.000 (9806582.940)	21340962.206 (5392854.224)	0.0051 (W)	0.0106	1.31	Up
49	AZELAIC ACID	80743.902 (55657.732)	48499.097 (19900.716)	0.0052 (W)	0.0106	1.66	Up
50	URIDINE	180401.501 (78579.325)	129485.672 (57369.706)	0.0067 (W)	0.0135	1.39	Up
51	4-METHYL-2-OXOVALERIC ACID	8259.714 (5554.566)	5424.737 (2564.106)	0.0070 (W)	0.0138	1.52	Up
52	LL-2-6-DIAMINOHEPTANEDIOATE.1	1723.832 (1639.063)	714.056 (530.789)	0.0079 (W)	0.015	2.41	Up
53	HYPOTAURINE	1681.570 (862.075)	3117.147 (2349.444)	0.0079 (W)	0.015	-1.85	Down
54	XANTHOSINE	1251.850 (1403.116)	667.877 (866.066)	0.0084 (W)	0.0156	1.87	Up
55	INDOXYL SULFATE	1696564.633 (1250913.918)	1124555.885 (853275.111)	0.0097 (W)	0.0178	1.51	Up
56	4-HYDROXY-2-QUINOLINECARBOXYLIC ACID	1431.589 (2196.795)	560.116 (809.237)	0.0106 (W)	0.0191	2.56	Up
57	D-PANTOTHENIC ACID	8410.794 (6212.275)	5248.162 (4216.996)	0.0110 (W)	0.0194	1.6	Up
58	R-MALATE	834.994 (746.458)	601.020 (744.681)	0.0114 (W)	0.0195	1.39	Up
59	BENZYL ALCOHOL	14603.304 (9896.197)	9359.557 (4700.703)	0.0219 (W)	0.0367	1.56	Up
60	L-CYSTATHIONINE	948.147 (1856.401)	357.815 (485.308)	0.0259 (W)	0.0428	2.65	Up
61	GUANIDINOACETATE	4741.159 (1649.526)	3944.362 (2014.868)	0.0291 (W)	0.0472	1.2	Up
62	N-METHYL-D-ASPARTIC ACID	282501.203 (102414.996)	405298.529 (241829.140)	0.0301 (W)	0.0481	-1.43	Down
63	5-OXO-L-PROLINE	1086282.943 (464545.366)	871105.550 (342599.621)	0.0358 (W)	0.0554	1.25	Up
64	2-AMINO-2-METHYLPROPANOATE	2429.040 (1022.633)	2047.369 (1386.439)	0.0358 (W)	0.0554	1.19	Up
65	SUCROSE	1244.689 (1641.116)	3885.591 (4356.109)	0.0382 (W)	0.0582	-3.12	Down
66	UROCANATE	19873.941 (8198.658)	16364.306 (11503.896)	0.0438 (W)	0.0656	1.21	Up
67	LAUROYLCARNITINE	859.315 (424.332)	680.869 (431.027)	0.0482 (W)	0.0702	1.26	Up
68	GLUTARATE	55463.211 (47870.540)	54087.955 (77000.886)	0.0482 (W)	0.0702	1.03	Up

Table S5. Unpaired t-test analysis of HILIC-MS dataset showing significant ($p < 0.05$) metabolites between civilian sarcoidosis cohort and COPD. (Highlight shows metabolites with FDR < 0.05).

	Name	Mean (SD) of COPD	Mean (SD) of Veteran sarcoidosis	p-value	q-value (FDR)	Fold Change	Veteran/COPD
1	3-ALPHA-11-BETA-17-ALPHA-21-TETRAHYDROXY- 5-ALPHA-PREGNAN-20-ONE	1825.514 (1191.736)	3501.975 (1568.464)	0.0003	0.0093	-1.92	UP
2	HYPOXANTHINE	308051.004 (188517.333)	519286.500 (177904.605)	0.0011	0.022	-1.69	UP
3	PALMITATE	4691.507 (1767.054)	2904.474 (1287.292)	0.0018	0.0302	1.62	Down
4	DEOXYCHOLATE	187962.614 (104105.216)	299856.992 (108606.372)	0.0021	0.0306	-1.6	Up
5	4-METHYL-2-OXO-PENTANOIC ACID	691326.344 (244980.000)	958995.600 (336473.667)	0.0042	0.0529	-1.39	Up
6	N-FORMYLGLYCINE	18921.098 (7001.746)	34902.312 (15836.197)	0.0002 (W)	0.0093	-1.84	Up
7	CHOLATE	28010.294 (16041.232)	52516.344 (20551.536)	0.0003 (W)	0.0093	-1.87	Up
8	TRANS-4-HYDROXYPROLINE	28751.865 (14461.930)	41794.125 (14818.436)	0.0010 (W)	0.022	-1.45	Up
9	CREATINE	40920.300 (31912.284)	54148.120 (23643.809)	0.0086 (W)	0.096	-1.32	Up
10	L-VALINE	19209.066 (17252.406)	22592.352 (12503.570)	0.0125 (W)	0.1137	-1.18	Up
11	SARCOSINE	69167.648 (51761.680)	87270.276 (38643.854)	0.0190 (W)	0.1587	-1.26	Up
12	N-METHYL-D-ASPARTIC ACID	405298.529 (241829.140)	567440.446 (259325.433)	0.0248 (W)	0.1832	-1.4	Up
13	SUBERIC ACID	30310.815 (15903.257)	20756.387 (7616.248)	0.0283 (W)	0.1832	1.46	Down
14	R-MALATE	601.020 (744.681)	812.285 (656.385)	0.0301 (W)	0.1832	-1.35	Up
15	L-THREONINE	229896.053 (70962.737)	279028.931 (80497.992)	0.0321 (W)	0.1832	-1.21	Up
16	L-METHIONINE	16612.641 (10795.283)	18724.125 (5671.912)	0.0321 (W)	0.1832	-1.13	Up
17	INOSINE 5-PHOSPHATE	1365.551 (2217.659)	2747.650 (2712.225)	0.0349 (W)	0.1835	-2.01	Up
18	XANTHOSINE	667.877 (866.066)	1014.593 (706.487)	0.0390 (W)	0.1888	-1.52	Up
19	MANNOSE	8266237.706 (2705069.100)	9570963.615 (2075416.765)	0.0396 (W)	0.1888	-1.16	Up
20	N-METHYL-L-GLUTAMATE	4207.634 (2558.014)	5376.967 (2343.073)	0.0435 (W)	0.1921	-1.28	Up
21	NALPHA-ACETYL-L-LYSINE	3574.632 (792.437)	4009.379 (731.646)	0.0461 (W)	0.1921	-1.12	Up
22	PROPIONATE	27543.556 (20756.470)	36747.736 (15794.559)	0.0489 (W)	0.1955	-1.33	Up

Table S6. Unpaired t-test analysis of HILIC-MS dataset showing significant ($p < 0.05$) metabolites between veteran sarcoidosis cohort and COPD. (Highlight shows metabolites with FDR < 0.05).

	Name	Mean (SD) of Sarcoidosis (Civilian)	Mean (SD) of Sarcoidosis (Veteran)	p-value	q-value (FDR)	Fold Change	Sarcoidosis (Civilian)/Sarcoidosis (Veteran)
1	**Rb85 c/s**	438216.833 (69566.532)	507826.892 (87277.551)	0.008	0.0311	-1.16	Down
2	**Sb121 c/s**	5385.907 (1852.773)	4717.231 (1705.446)	0.2726	0.3816	1.14	Up
3	**Rh103 c/s**	119.433 (27.650)	124.923 (32.771)	0.5749	0.6477	-1.05	Down
4	**Sc45 c/s**	146347.313 (37125.916)	119326.062 (10974.504)	0.0003 (W)	0.0084	1.23	Up
5	**Cr52 c/sICRC**	882.067 (334.552)	1079.692 (198.422)	0.0005 (W)	0.0084	-1.22	Down
6	**Se77 c/s ICRC**	704.300 (232.938)	473.000 (140.344)	0.0008 (W)	0.0096	1.49	Up
7	**Ga71 c/s**	18388.733 (57103.712)	16223.277 (22712.195)	0.0027 (W)	0.0235	1.13	Up
8	**Au197 c/s**	659.933 (228.966)	496.462 (215.608)	0.0039 (W)	0.0249	1.33	Up
9	**Mg24 c/s ICRC**	289118.587 (160481.059)	325388.123 (53005.934)	0.0043 (W)	0.0249	-1.13	Down
10	**Al27 c/s**	483132.423 (599395.237)	696144.923 (560721.719)	0.0056 (W)	0.0281	-1.44	Down
11	**Ti49 c/s**	4207.460 (2690.849)	4223.400 (851.857)	0.0080 (W)	0.0311	-1	Down
12	**As75 c/s ICRC**	643.397 (2467.610)	1029.339 (1088.356)	0.0103 (W)	0.036	-1.6	Down
13	**Mn55 c/s**	169399.773 (267089.702)	173363.138 (139822.547)	0.0193 (W)	0.0606	-1.02	Down
14	**Se78 c/s ICRC**	557.268 (113.913)	476.156 (124.139)	0.0208 (W)	0.0606	1.17	Up
15	**Hg202 c/s**	1045.167 (384.337)	818.231 (75.256)	0.0272 (W)	0.0733	1.28	Up
16	**Ba137 c/s**	4471.740 (9102.251)	4669.585 (3943.251)	0.0333 (W)	0.0801	-1.04	Down
17	**Ca44 c/s ICRC**	48222.540 (12419.501)	54640.954 (7514.699)	0.0343 (W)	0.0801	-1.13	Down
18	**Fe56 c/s ICRC**	32606.833 (24937.991)	37108.262 (12441.659)	0.0449 (W)	0.0982	-1.14	Down

Table S7. Unpaired t-test analysis of individual elements detected by ICP-MS between veterans and civilians with confirmed sarcoidosis. Elements highlighted in yellow have a FDR <0.05.

	Name	Mean (SD) of COPD	Mean (SD) of Sarcoidosis (Civilian)	p-value	q-value (FDR)	Fold Change	COPD/Sarcoidosis (Civilian)
1	Rb85 c/s	507258.954 (82266.687)	438216.833 (69566.532)	0.0006	0.0013	1.16	Up
3	Se78 c/s ICRC	345.529 (60.993)	557.268 (113.913)	< 0.0001	0	-1.61	Down
4	B11 c/s	22638.446 (3142.308)	43115.893 (53016.162)	< 0.0001 (W)	0	-1.9	Down
5	Al27 c/s	488515.994 (283641.188)	483132.423 (599395.237)	< 0.0001 (W)	0	1.01	Up
6	Sc45 c/s	99619.006 (4099.973)	146347.313 (37125.916)	< 0.0001 (W)	0	-1.47	Down
7	Mn55 c/s	111931.537 (32038.741)	169399.773 (267089.702)	< 0.0001 (W)	0	-1.51	Down
8	Ga71 c/s	5131.966 (6140.210)	18388.733 (57103.712)	< 0.0001 (W)	0	-3.58	Down
9	Sb121 c/s	3674.749 (1508.522)	5385.907 (1852.773)	< 0.0001 (W)	0	-1.47	Down
10	Au197 c/s	272.914 (35.077)	659.933 (228.966)	< 0.0001 (W)	0	-2.42	Down
11	Hg202 c/s	707.886 (57.711)	1045.167 (384.337)	< 0.0001 (W)	0	-1.48	Down
12	Mg24 c/s ICRC	321335.029 (30235.816)	289118.587 (160481.059)	< 0.0001 (W)	0.0001	1.11	Up
13	Ca44 c/s ICRC	58898.560 (4159.104)	48222.540 (12419.501)	< 0.0001 (W)	0	1.22	Up
14	Fe56 c/s ICRC	50479.834 (44767.652)	32606.833 (24937.991)	< 0.0001 (W)	0.0001	1.55	Up
15	As75 c/s ICRC	814.607 (377.567)	643.397 (2467.610)	< 0.0001 (W)	0	1.27	Up
16	Se77 c/s ICRC	277.086 (50.658)	704.300 (232.938)	< 0.0001 (W)	0	-2.54	Down
17	Cr52 c/sICRC	1193.829 (279.369)	882.067 (334.552)	< 0.0001 (W)	0	1.35	Up
18	Pt195 c/s	24.486 (5.170)	1063.540 (5619.057)	0.0012 (W)	0.0024	-43.44	Down
19	Ba137 c/s	3369.269 (2669.586)	4471.740 (9102.251)	0.0015 (W)	0.0029	-1.33	Down
20	Ti49 c/s	3913.091 (437.889)	4207.460 (2690.849)	0.0024 (W)	0.0044	-1.08	Down
21	Pr141 c/s	10500.869 (37297.105)	1677.860 (4373.640)	0.0092 (W)	0.016	6.26	Up
22	Nd146 c/s	5848.794 (20851.774)	855.067 (2188.974)	0.0117 (W)	0.019	6.84	Up
23	La139 c/s	120814.131 (421974.513)	32798.453 (95172.133)	0.0120 (W)	0.019	3.68	Up
24	Ni60 c/s	74938.920 (208236.904)	176629.433 (490496.189)	0.0217 (W)	0.033	-2.36	Down
25	Ce140 c/s	173426.423 (603807.625)	63597.760 (190431.346)	0.0376 (W)	0.0534	2.73	Up
26	Gd157 c/s	214.771 (666.592)	300.833 (682.285)	0.0381 (W)	0.0534	-1.4	Down

Table S8, Unpaired t-test analysis of metallome elements between civilian subjects with sarcoidosis and veterans with COPD

	Name	Mean (SD) of COPD	Mean (SD) of Sarcoidosis (Veteran)	p-value	q-value (FDR)	Fold Change	COPD/Sarcoidosis (Veteran)
1	**Sc45 c/s**	99619.006 (4099.973)	119326.062 (10974.504)	< 0.0001 (W)	0	-1.2	Down
2	**Au197 c/s**	272.914 (35.077)	496.462 (215.608)	< 0.0001 (W)	0	-1.82	Down
3	**Hg202 c/s**	707.886 (57.711)	818.231 (75.256)	< 0.0001 (W)	0.0004	-1.16	Down
8	**Se78 c/s ICRC**	345.529 (60.993)	476.156 (124.139)	< 0.0001 (W)	0.0005	-1.38	Down
9	**Se77 c/s ICRC**	277.086 (50.658)	473.000 (140.344)	< 0.0001 (W)	0	-1.71	Down
10	**Pt195 c/s**	24.486 (5.170)	33.231 (6.405)	0.0003 (W)	0.0015	-1.36	Down
11	**Cs133 c/s**	2501.200 (681.532)	5390.554 (5804.694)	0.0004 (W)	0.0022	-2.16	Down
12	**B11 c/s**	22638.446 (3142.308)	31077.862 (13931.954)	0.0006 (W)	0.0026	-1.37	Down
13	**Gd157 c/s**	214.771 (666.592)	2386.415 (8328.709)	0.0110 (W)	0.043	-11.11	Down
14	**Sb121 c/s**	3674.749 (1508.522)	4717.231 (1705.446)	0.0267 (W)	0.0935	-1.28	Down

Table S9, Unpaired t-test analysis of metallome elements between veterans with sarcoidosis and veterans with COPD

Name	Mean (SD) of Stage 123	Mean (SD) of Stage 4	p-value	q-value (FDR)	Fold Change	Stage 123/Stage 4
CHOLATE	60098. (20753)	42674 (20364)	0.0193	0.6274	1.41	Up
5-OXO-L-PROLINE	1014897 (282728)	1348250 (529887)	0.0511	0.6468	-1.33	Down
INOSINE 5-PHOSPHATE	2498 (1445)	1662 (2065)	0.0184 (W)	0.6274	1.5	Up
4-HYDROXYBENZALDEHYDE	54144 (10467)	47090 (8559)	0.0215 (W)	0.6274	1.15	Up
DOCOSAHEXAENOIC ACID	444425 (302390)	769144 (447414)	0.0359 (W)	0.6274	-1.73	Down
3-2-HYDROXYPHENYLPROPANOATE	36908 (55980)	66601 (70863)	0.0359 (W)	0.6274	-1.8	Down
S-DIHYDROOROTATE	1930 (1235)	2678 (1062)	0.0365 (W)	0.6274	-1.39	Down

Table S10. Unpaired t-test analysis showing significantly changed metabolites between sarcoidosis radiologic stage 4 and stages 1- 3. There is no FDR significant metabolites

Biochemical Pathways	Total	Expected	Hits	Raw p	= -Log P	Holm adjust	FDR	Impact
**Synthesis and degradation of ketone bodies**	6	0.08226	2	0.002643	5.936	0.1929	0.026425	0.7
**Arginine and proline metabolism**	77	1.0557	8	5.53E-06	12.105	0.000432	0.000148	0.42663
**Glycine, serine and threonine metabolism**	48	0.65808	5	0.000398	7.8288	0.029859	0.005308	0.4211
**Taurine and hypotaurine metabolism**	20	0.2742	2	0.029692	3.5169	1	0.16967	0.36331
**Alanine, aspartate and glutamate metabolism**	24	0.32904	4	0.000257	8.2675	0.019512	0.004108	0.31244
**beta-Alanine metabolism**	28	0.38388	2	0.055196	2.8969	1	0.24532	0.25694
**Histidine metabolism**	44	0.60324	1	0.45825	0.78035	1	0.96473	0.13988
**Pyruvate metabolism**	32	0.43872	1	0.35895	1.0246	1	0.84458	0.13756
**Propanoate metabolism**	35	0.47985	6	5.05E-06	12.195	0.000399	0.000148	0.12817
**Phenylalanine metabolism**	45	0.61695	2	0.12535	2.0766	1	0.43601	0.11906

Table S12. Pathway analysis from ^{1}H-NMR dataset

Biochemical Pathways	Total	Expected	Hits	Raw p	= -Log P	Holm adjust	FDR	Impact
**Taurine and hypotaurine metabolism**	20	0.26589	3	0.002088	6.1716	0.16077	0.041758	0.48202
**Alanine, aspartate and glutamate metabolism**	24	0.31907	4	0.000227	8.39	0.017716	0.006057	0.26401
**Glutathione metabolism**	38	0.50519	2	0.089517	2.4133	1	0.59561	0.23933
**Arginine and proline metabolism**	77	1.0237	4	0.01764	4.0376	1	0.23521	0.20375
**Phenylalanine metabolism**	45	0.59826	2	0.11912	2.1276	1	0.59561	0.11906
**Tryptophan metabolism**	79	1.0503	1	0.65868	0.41751	1	1	0.10853
**Nicotinate and nicotinamide metabolism**	44	0.58496	2	0.11475	2.165	1	0.59561	0.10565
**Glycerophospholipid metabolism**	39	0.51849	2	0.093594	2.3688	1	0.59561	0.10384

Table S13. Pathway analysis from HILIC-MS dataset

A

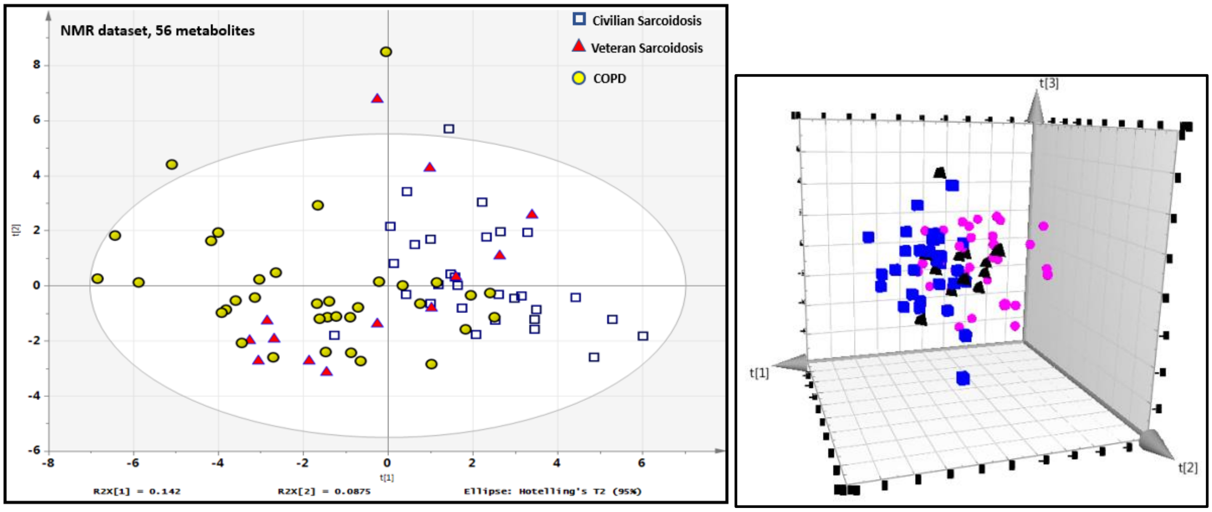

B

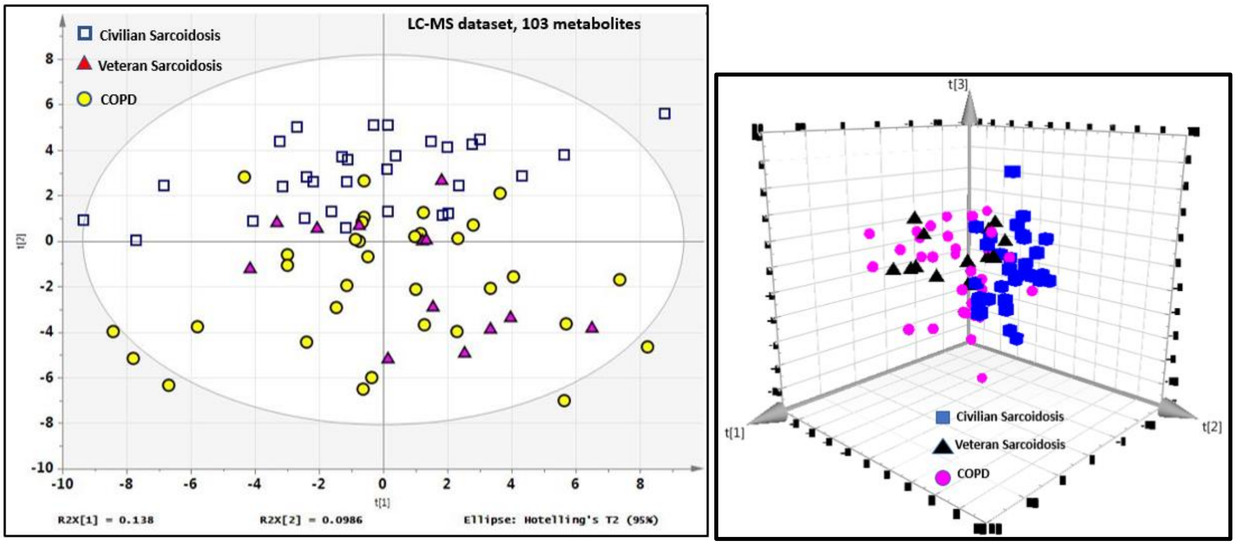

Figure S1. Principal component analysis (PCA) model metabolomic data obtained from the three studied cohorts by A. 1H-NMR and B. HILIC-MS.

A

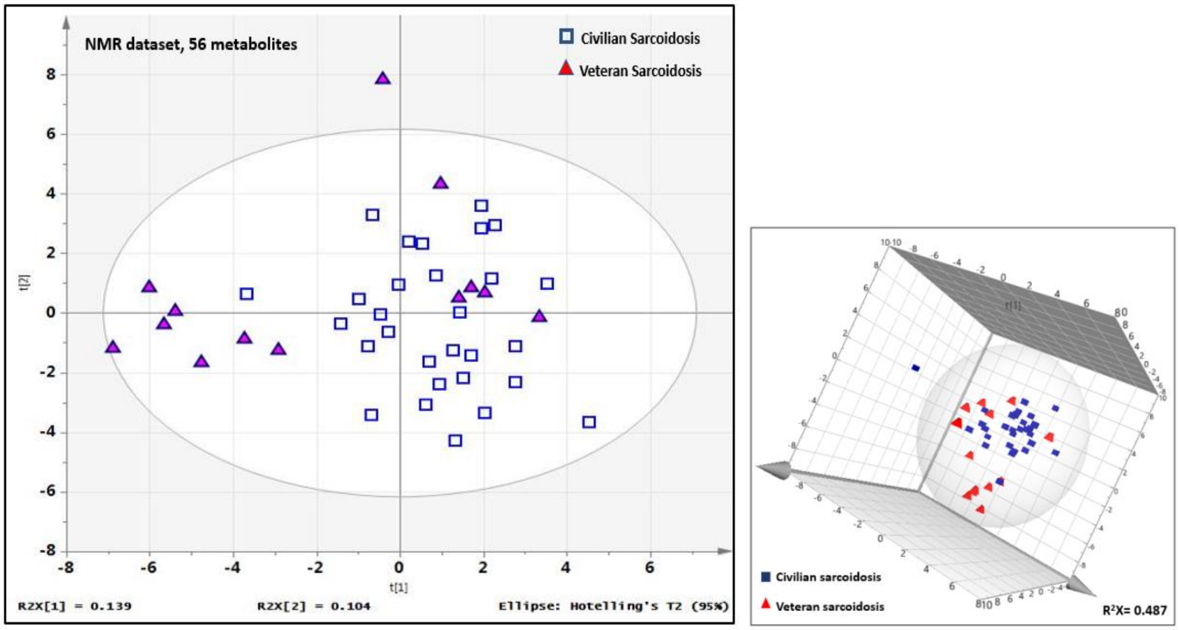

B

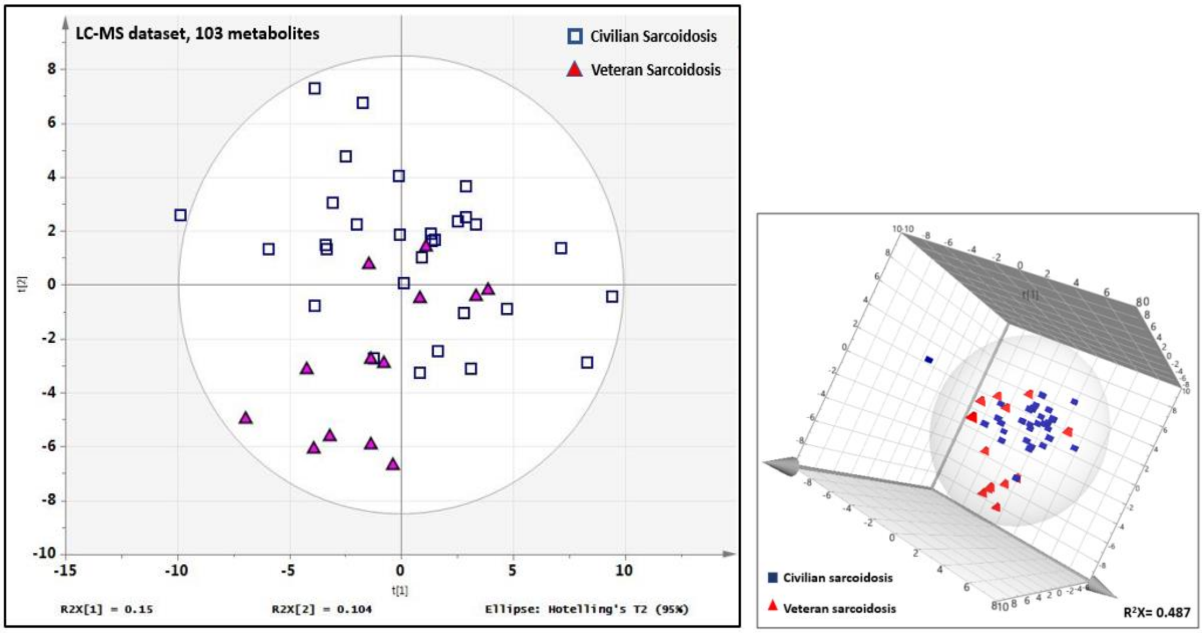

Figure S2. Principal component analysis (PCA) model metabolomic data comparing veterans and civilians with confirmed sarcoidosis, obtained by A. ^{1}H-NMR and B. HILIC-MS.

A

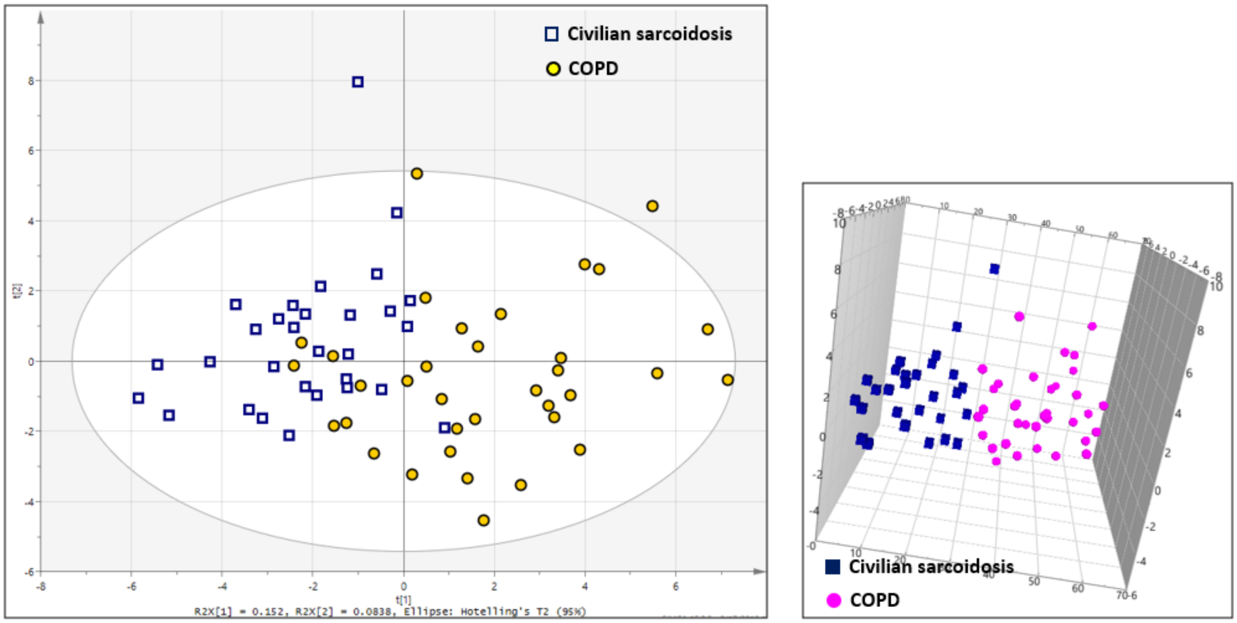

B

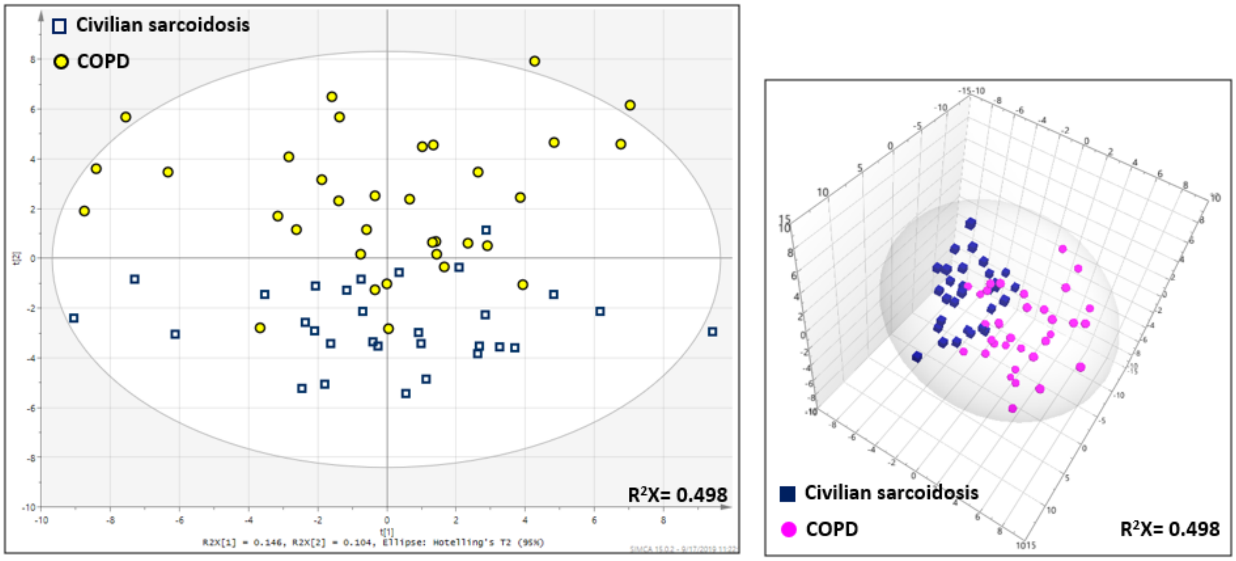

Figure S3. Principal component analysis (PCA) model metabolomic data comparing civilian sarcoidosis vs. COPD control, obtained by A. ^{1}H-NMR and B. HILIC-MS.

A

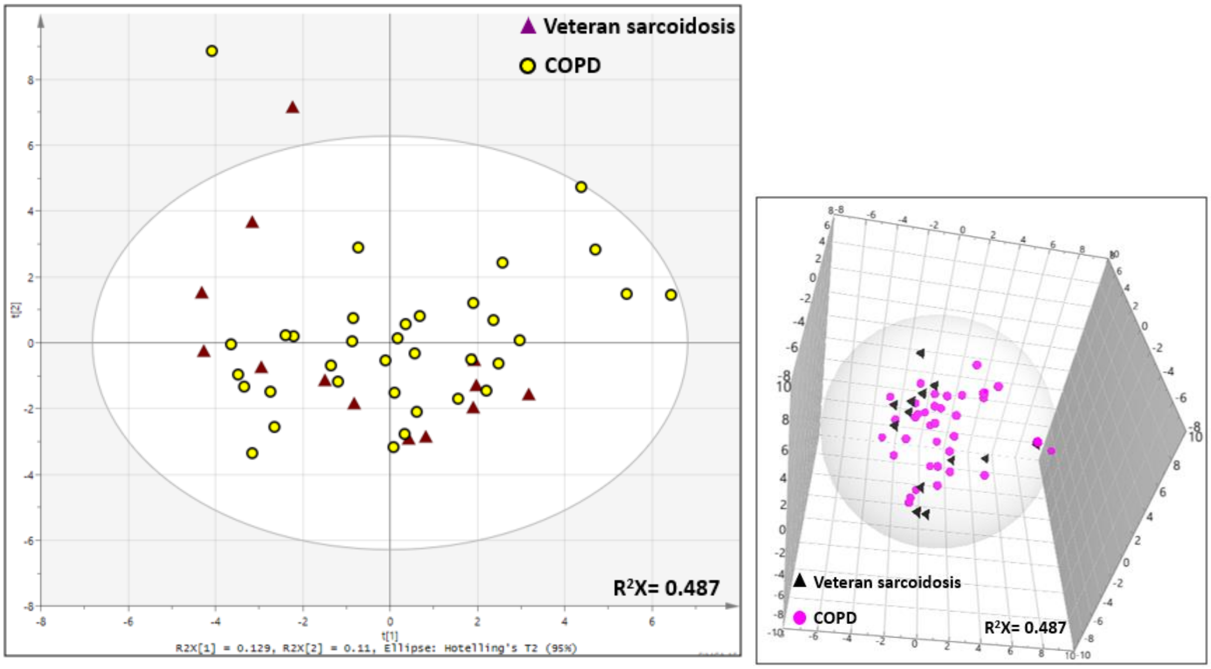

B

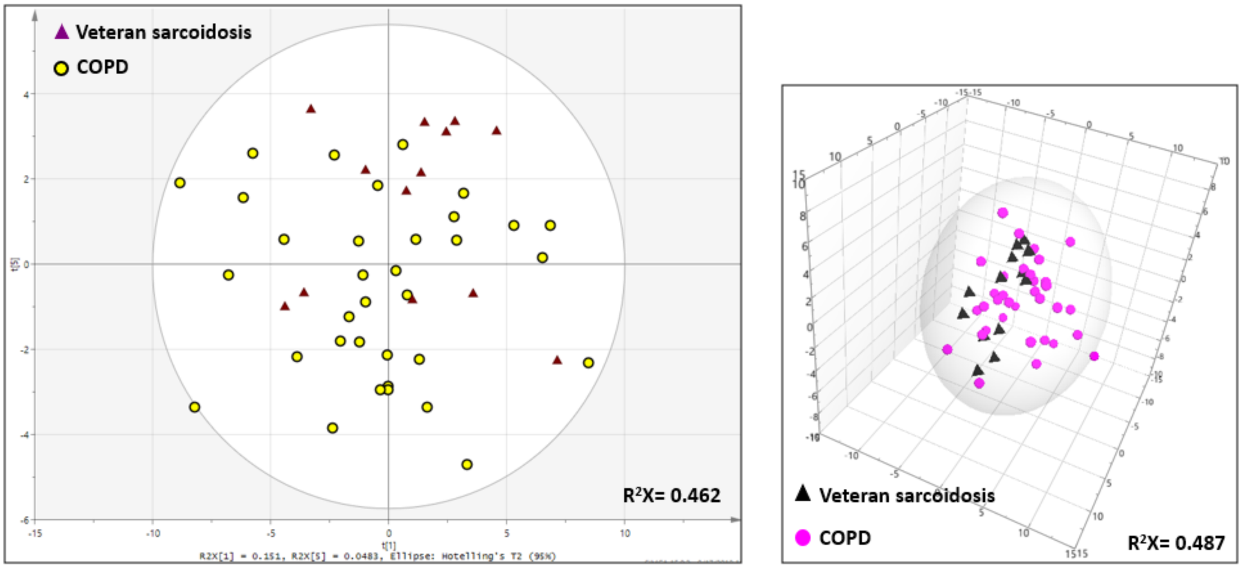

Figure S4. Principal component analysis (PCA) model metabolomic data comparing veteran sarcoidosis vs. COPD control, obtained by A. $_1$H-NMR and B. HILIC-MS.

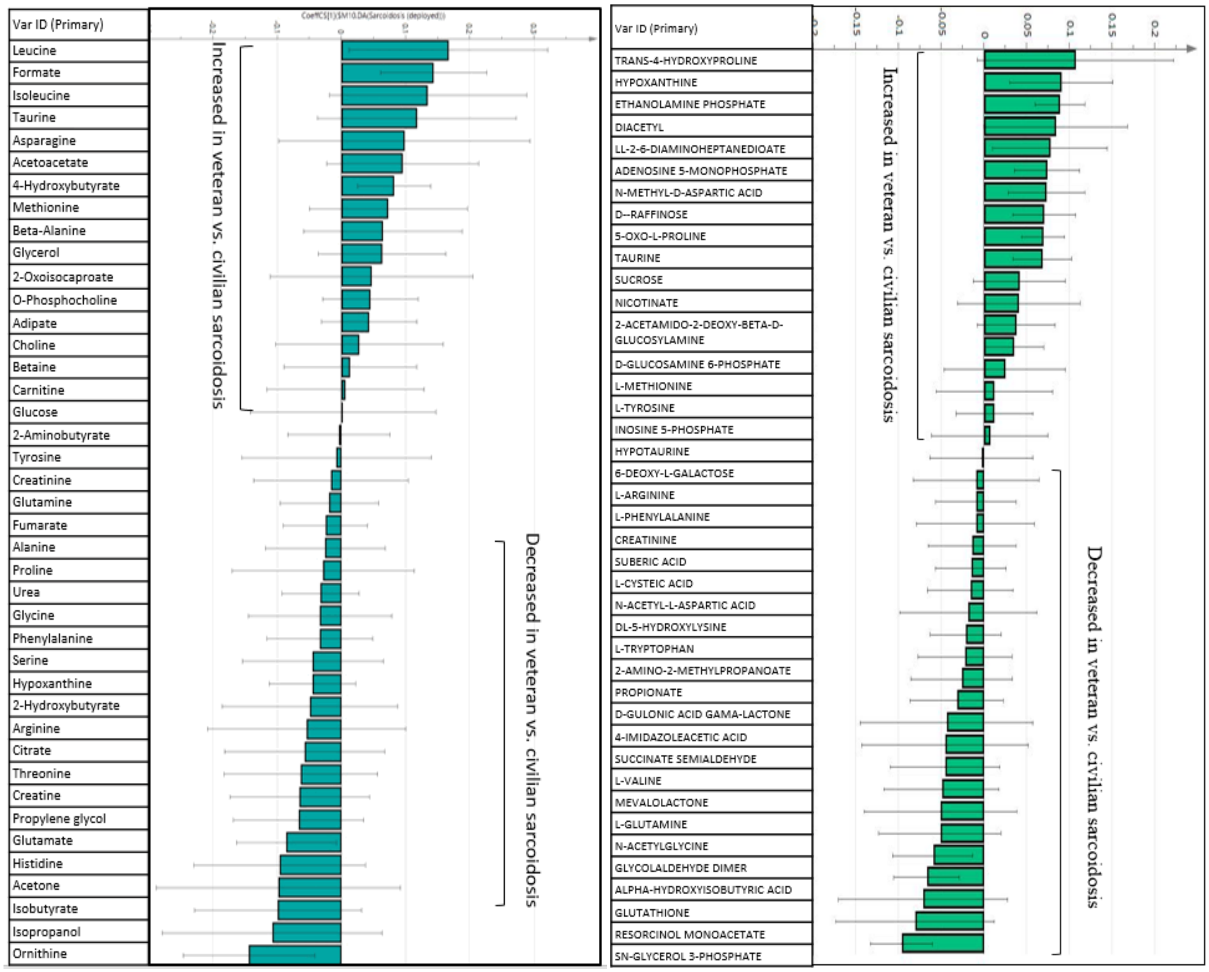

A B

Figure S5. Coefficient plot show the correlative relation of metabolites between veteran and civilian sarcoidosis. A. 1H-NMR dataset and B. the HILIC-MS dataset.

A

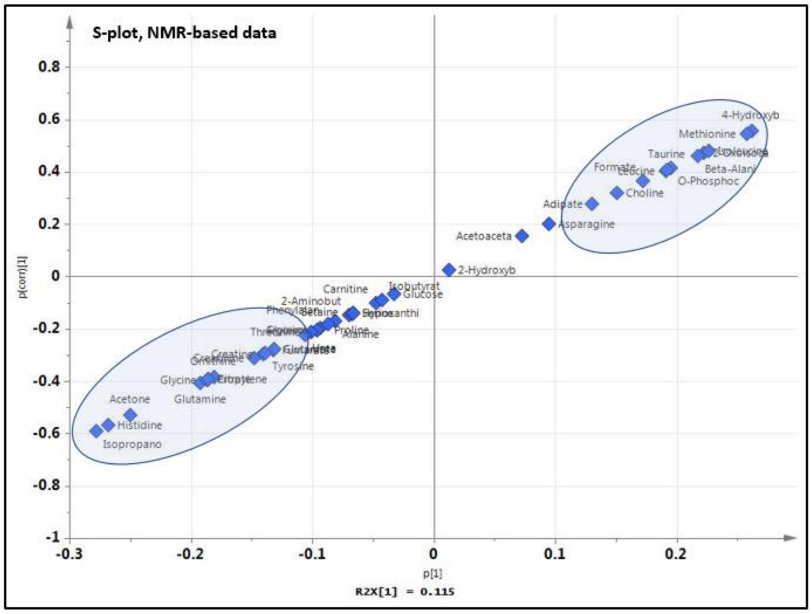

B.

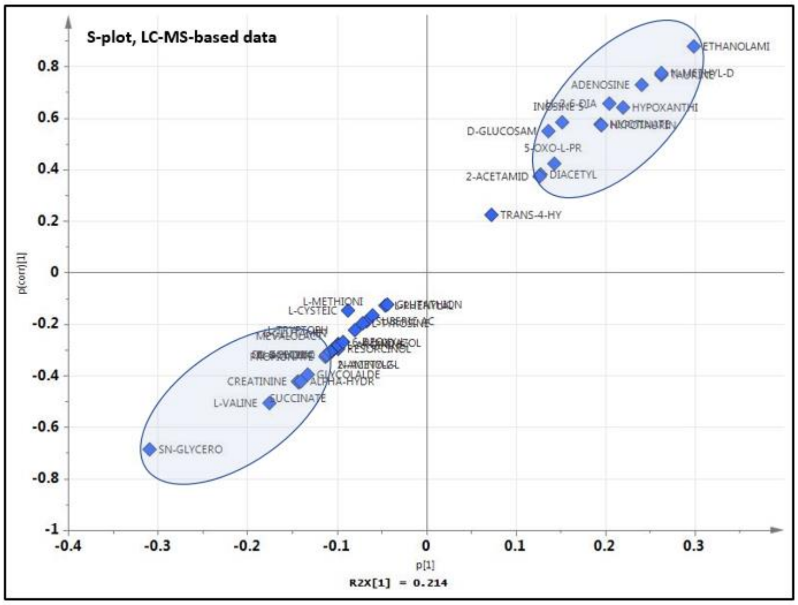

Figure S6, S-plot for metabolites detected by A. ^{1}H-NMR spectroscopy and B. HILIC-MS. Important metabolites with covariance more than 0.1 have been highlighted.

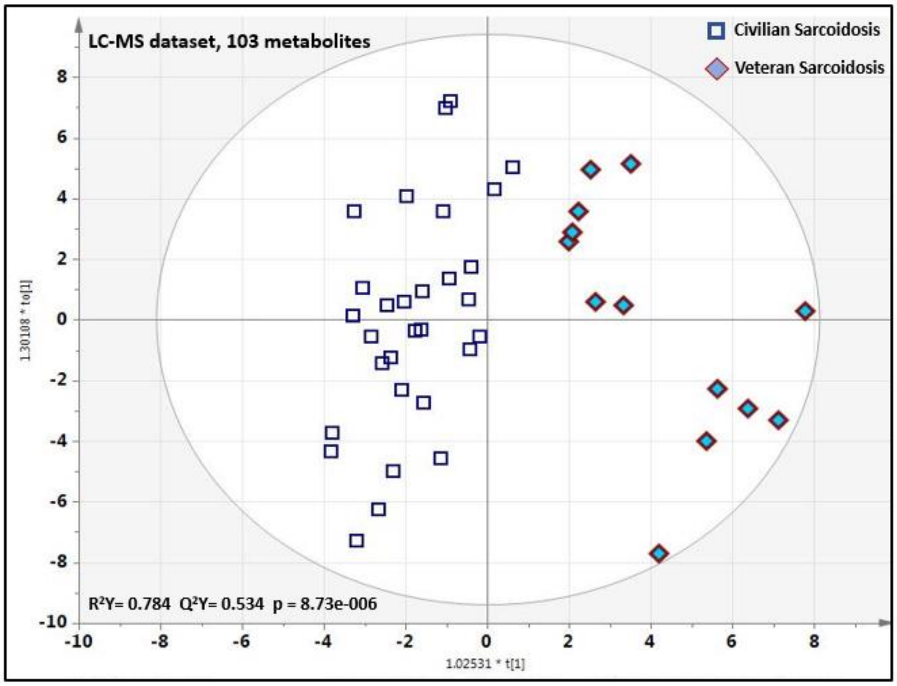

Figure S7, OPLS-DA of HILIC-MS- based metabolomics using all identified metabolites (n=103).

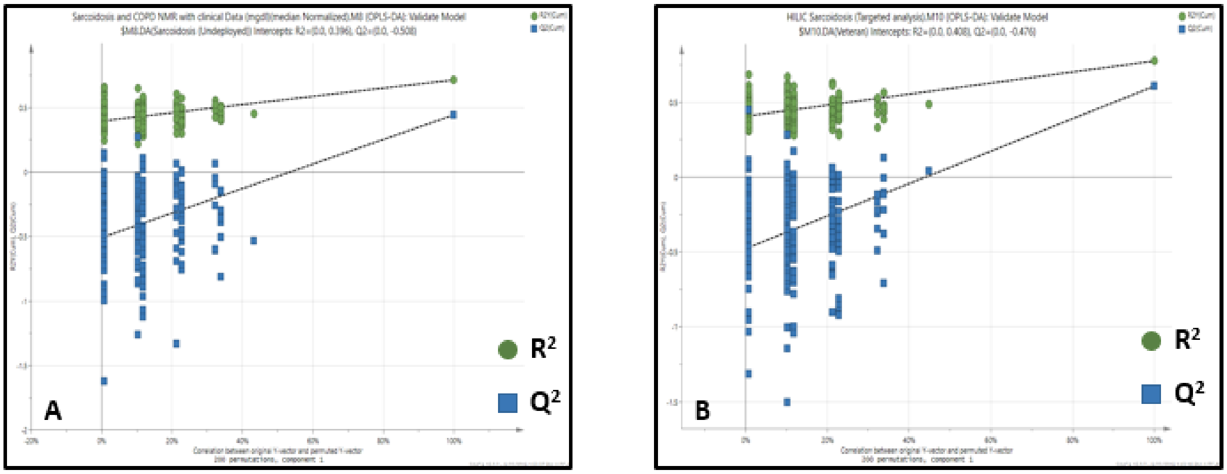

Figure S8, permutation tests (200 times repetitions) validated the goodness of fitness **(R^2)** and goodness of predictability **(Q^2)** to separate veteran cohort from civilian cohort with sarcoidosis. A: ^{1}H NMR dataset, B: HILIC-MS dataset

A B

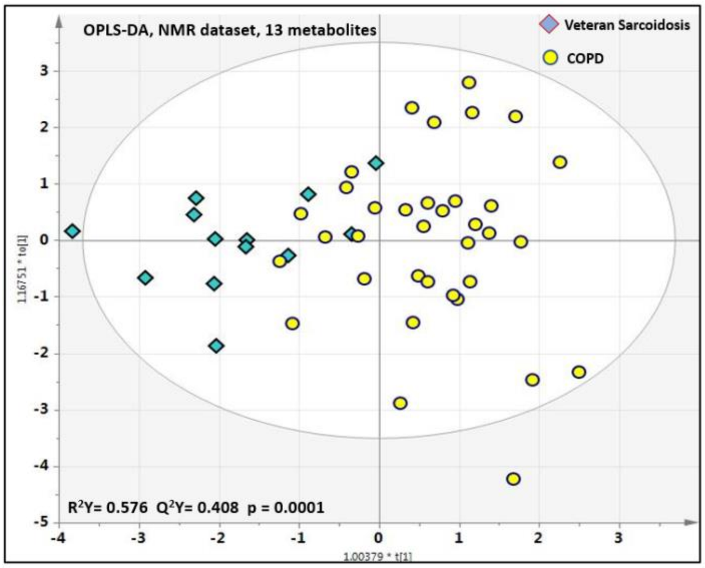

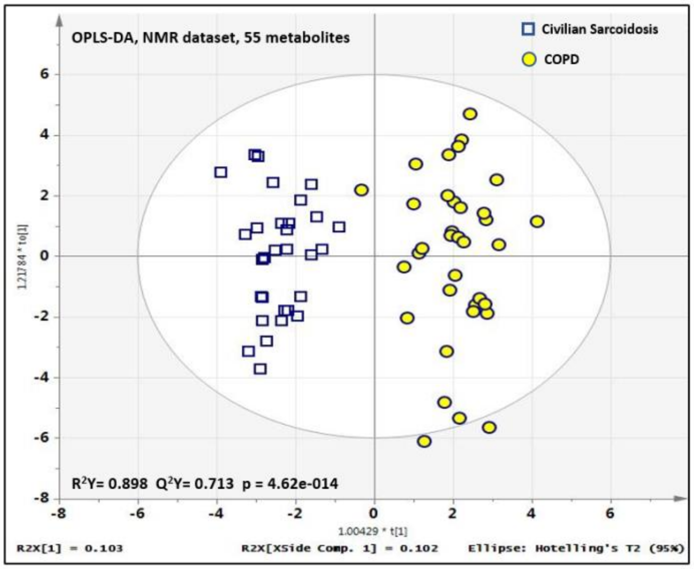

Figure S9, OPLS-DA model using 1H-NMR-based metabolomics to separate subjects with sarcoidosis from subjects with COPD. A. comparing veterans with COPD with veterans with sarcoidosis and B. Comparing veterans with COPD from civilians with sarcoidosis.

A B

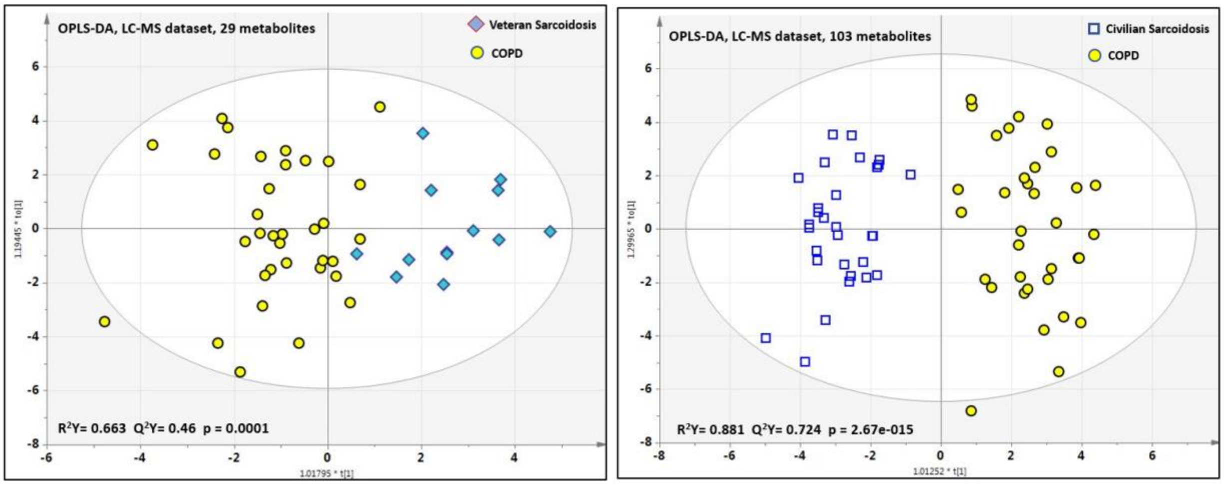

Figure S10. OPLS-DA model using HILIC-MS-based metabolomics to separate subjects with sarcoidosis from subjects with COPD. A. comparing veterans with COPD with veterans with sarcoidosis and B. Comparing veterans with COPD from civilians with sarcoidosis.

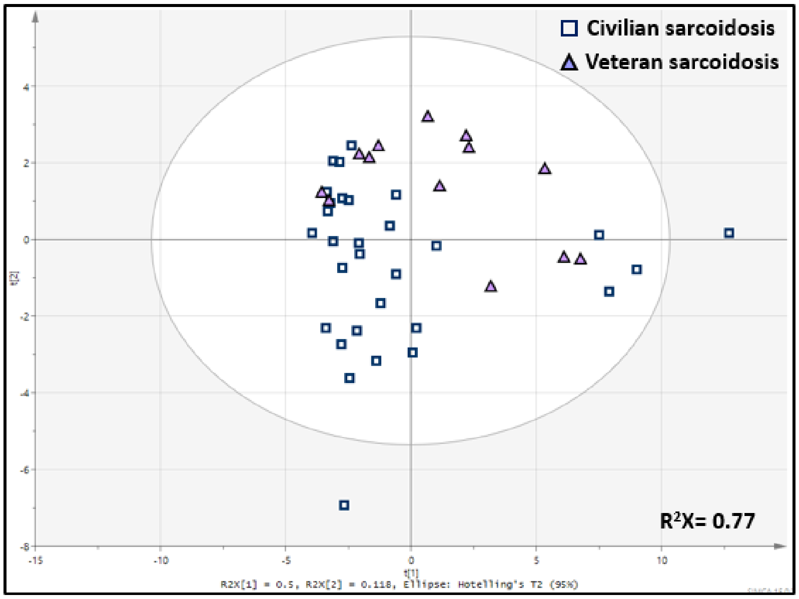

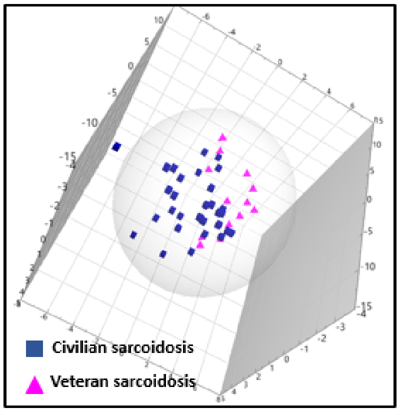

Figure S11. PCA model (2D and 3D plots) of metallomic data obtained by ICP-MS shows the contribution of 33 elements measured in separating veteran subjects with sarcoidosis from civilians with sarcoidosis

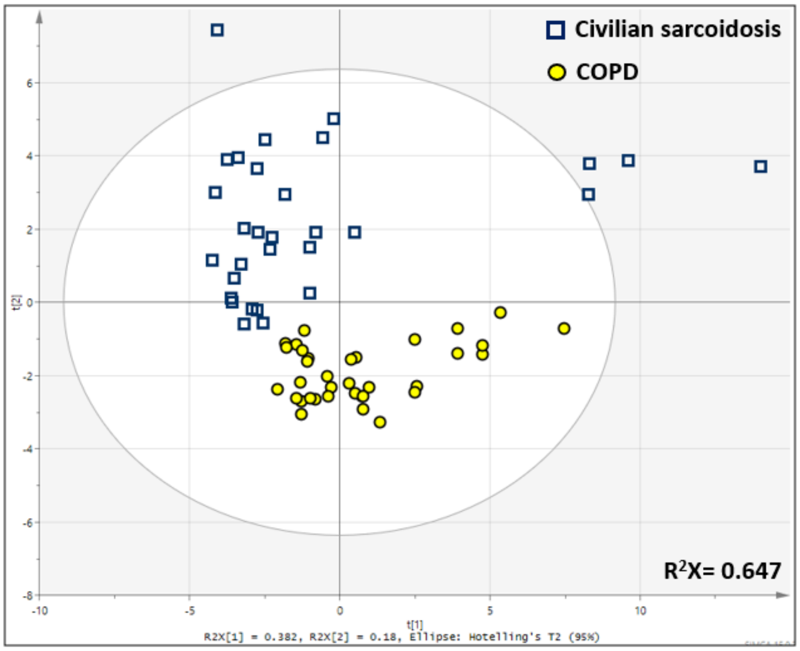

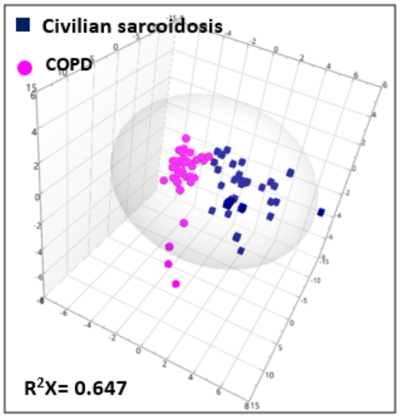

Figure S12. PCA model (2D and 3D plots) of metallomic data obtained by ICP-MS shows the contribution of 33 elements measured in separating civilian subjects with sarcoidosis from COPD controls.

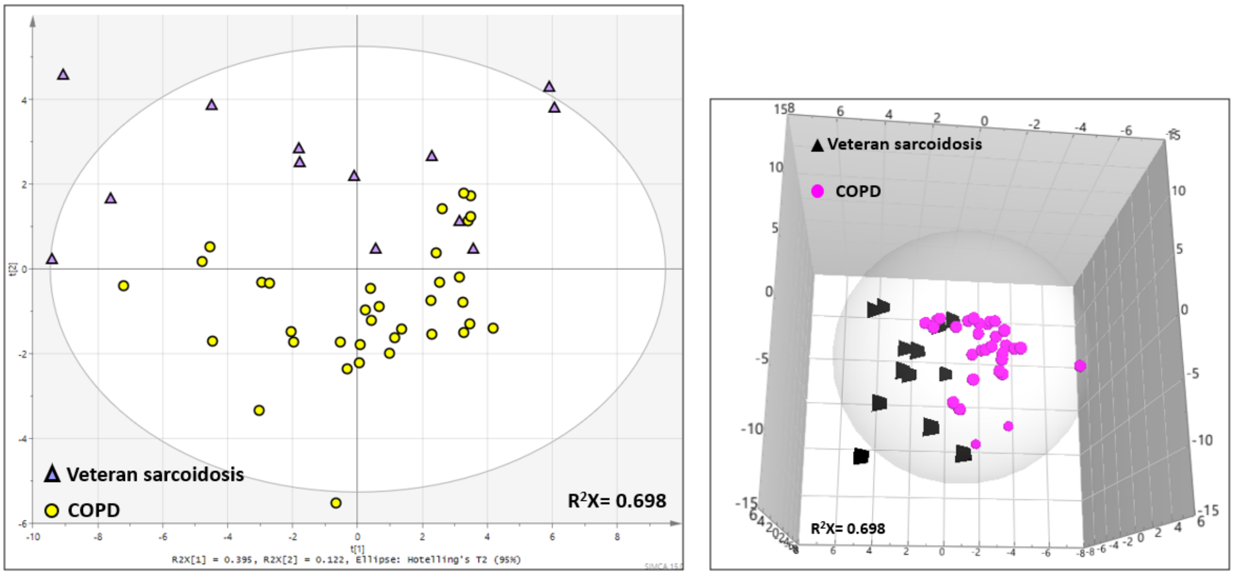

Figure S13. PCA model (2D and 3D plots) of metallomic data obtained by ICP-MS shows the contribution of 33 elements measured in separating veteran subjects with sarcoidosis from COPD controls.

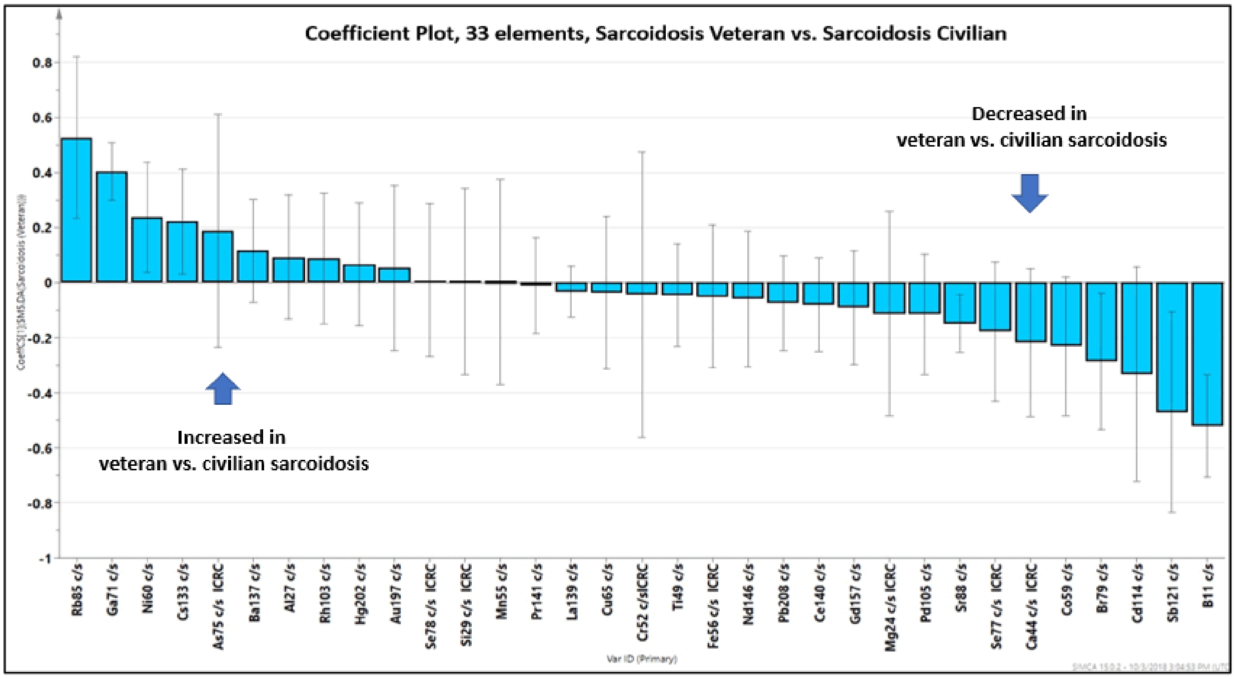

Figure S14. Coefficient plot shows alterations of 33 elements in veteran and civilians with confirmed sarcoidosis.

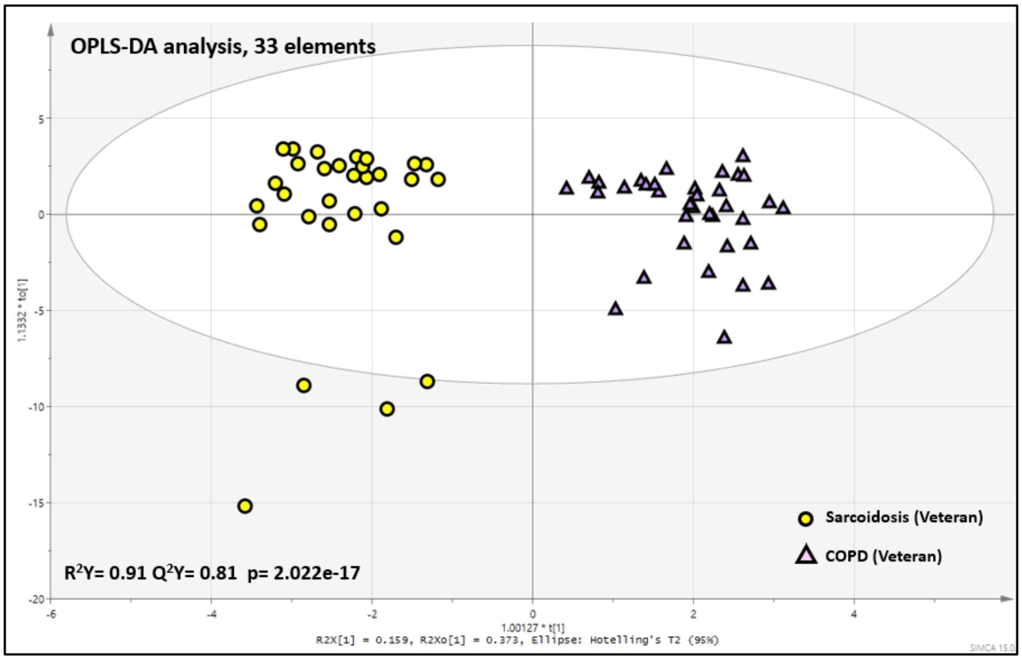

A

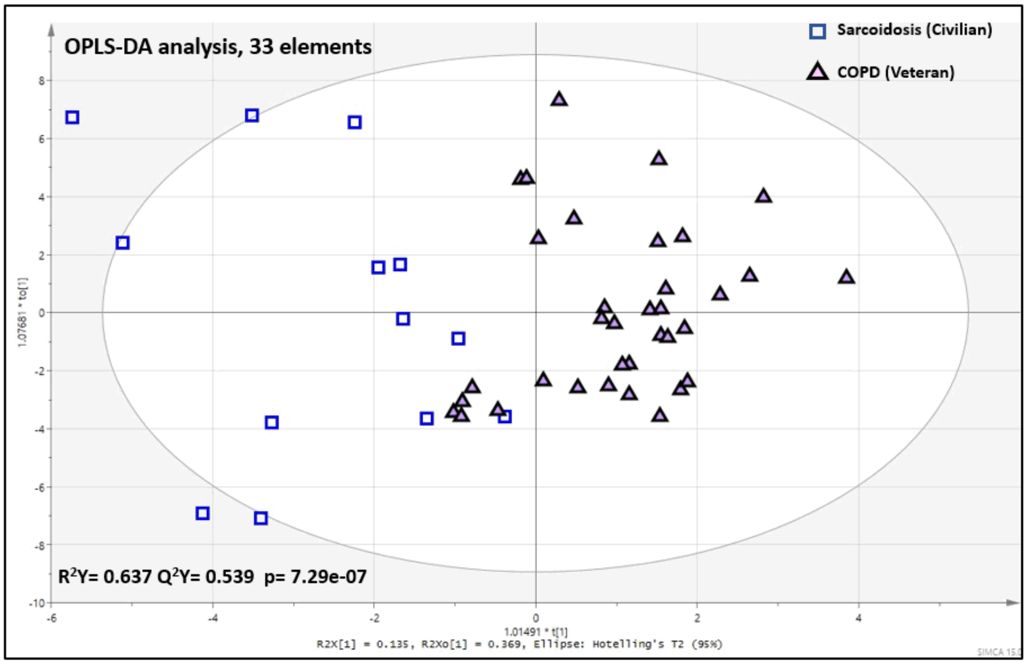

B

Figure S15, OPLS-DA of ICP-MS- based metallomics data using all identified metal ions comparing veterans with COPD with A. civilian subjects with sarcoidosis (n=33 elements) and B. veterans with sarcoidosis (N=33 elements).

A

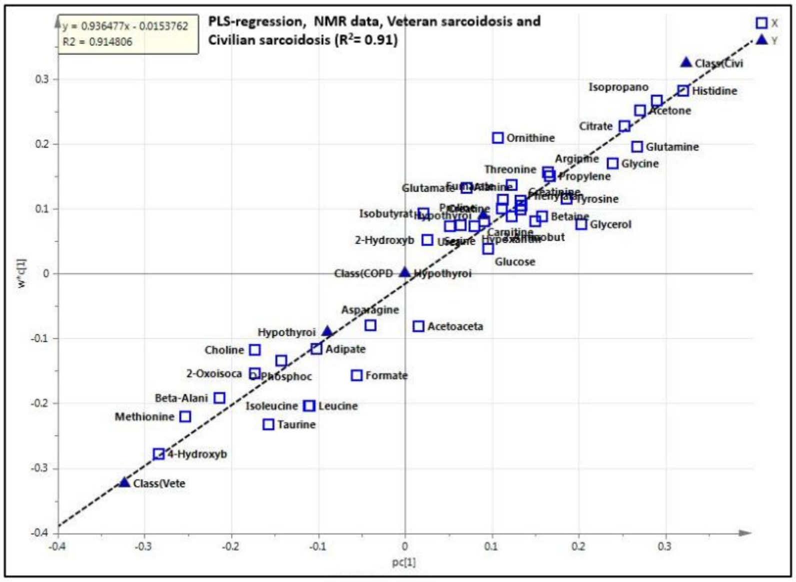

B

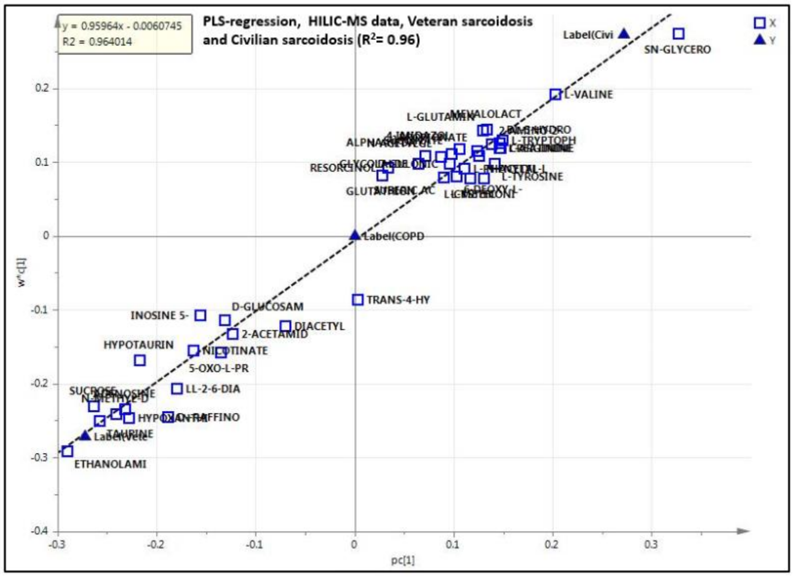

Figure S16. Partial least square regression (PLSR) analysis shows a strong relation between most important metabolites in relation to the sarcoidosis cohort type (veteran or civilian).
A. using metabolites detected by $_1$H-NMR. B. Using metabolites detected by HILIC-MS.

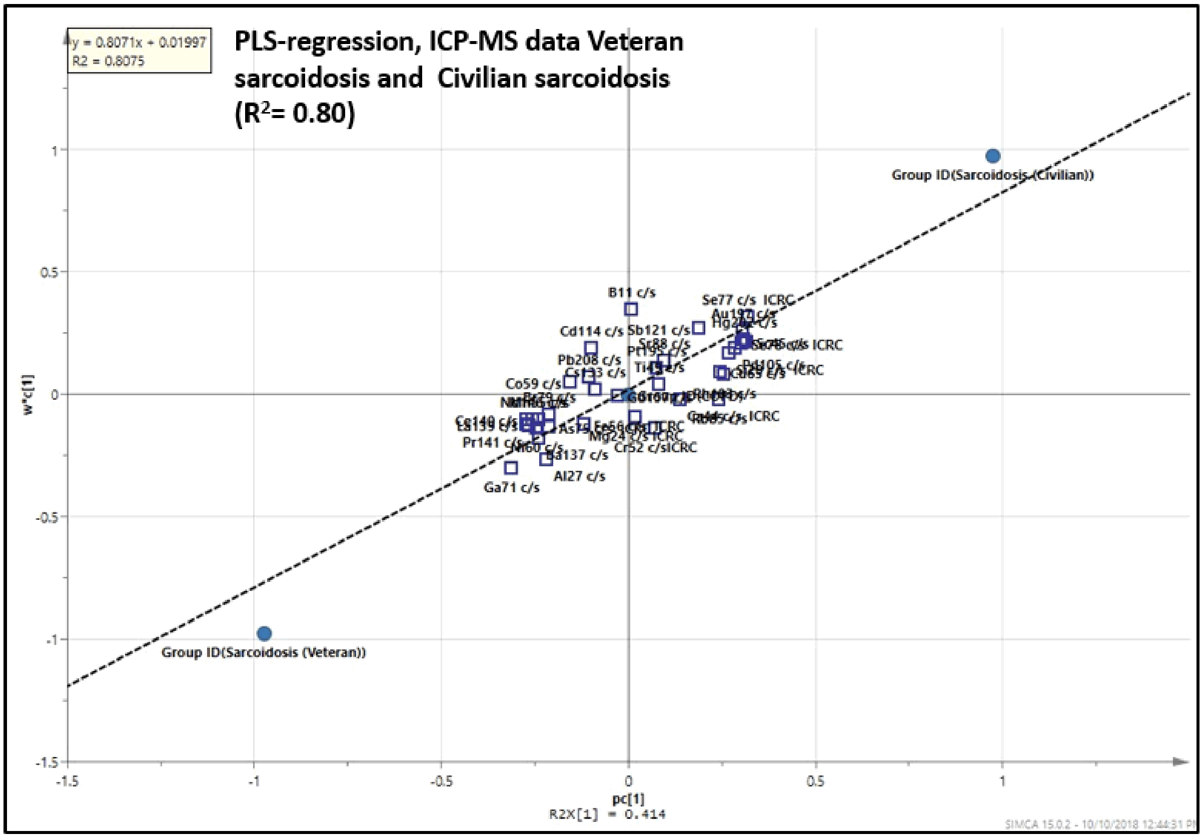

Figure S17. Partial least square regression (PLSR) analysis shows a relative strong relation between 33 elements detected by ICP-MS in relation to the sarcoidosis cohort type (veteran or civilian).

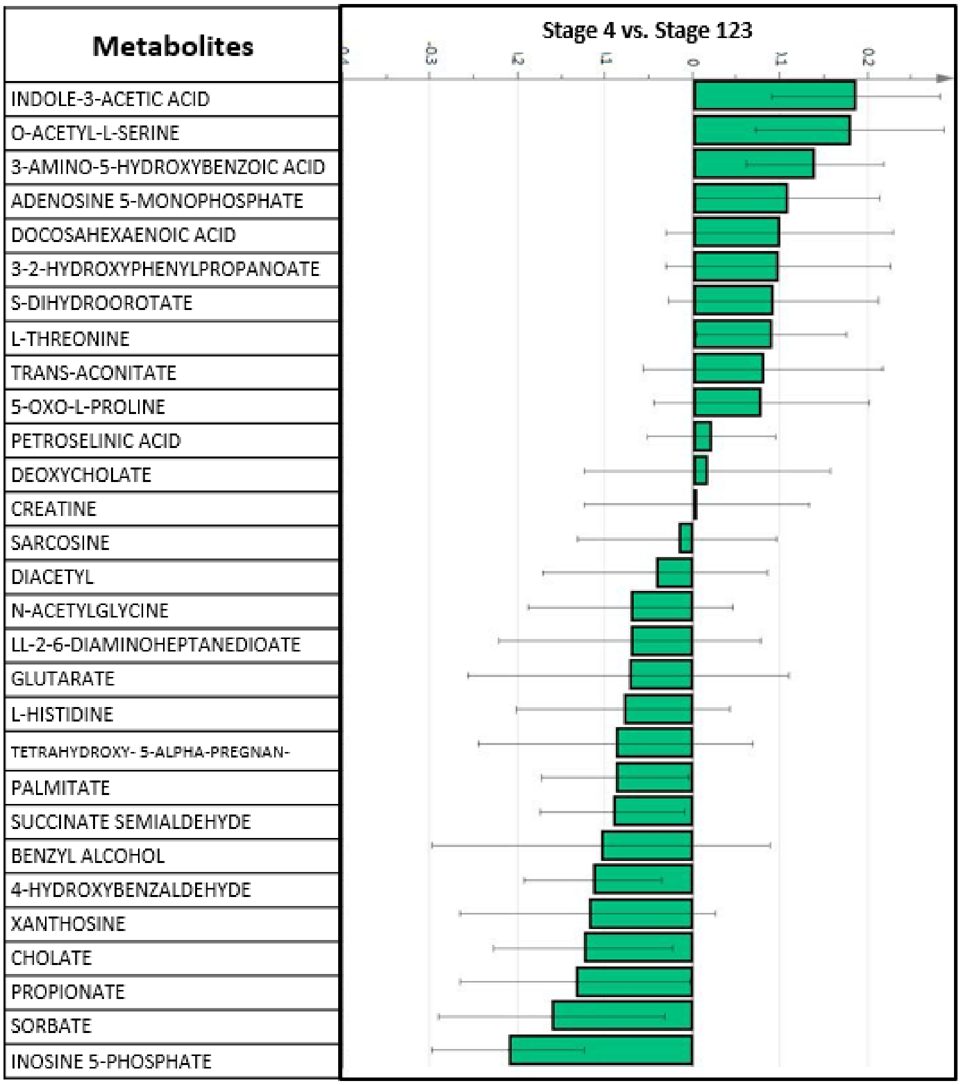

Figure S18. Coefficient plot shows relative correlation of measured metabolites between subjects with sarcoidosis radiologic stage 4 versus subjects with stages 1-3.

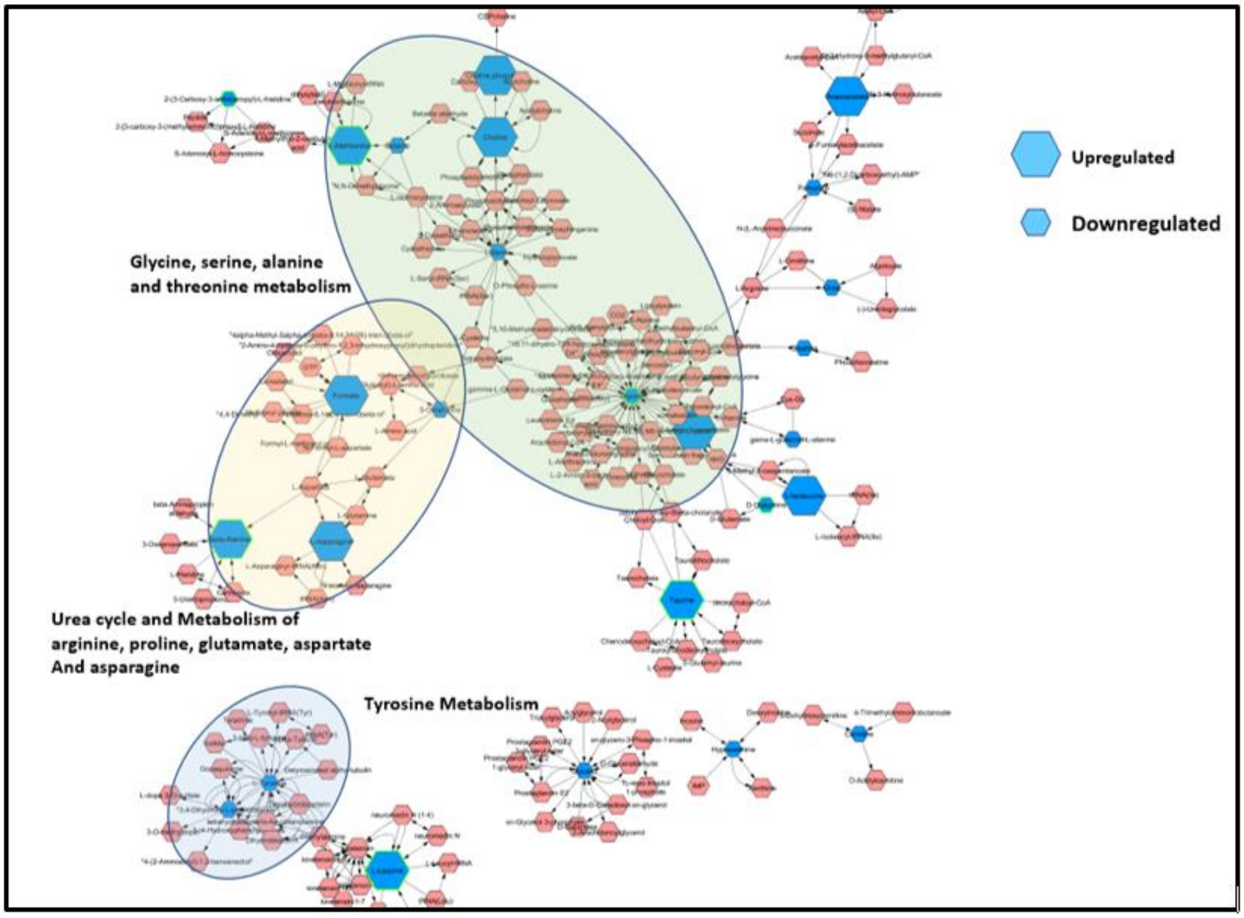

Figure S14, Cytoscape-based pathway analysis using ^{1}H-NMR data showing several metabolic pathways that differ between veteran sarcoidosis and civilian sarcoidosis cohorts.

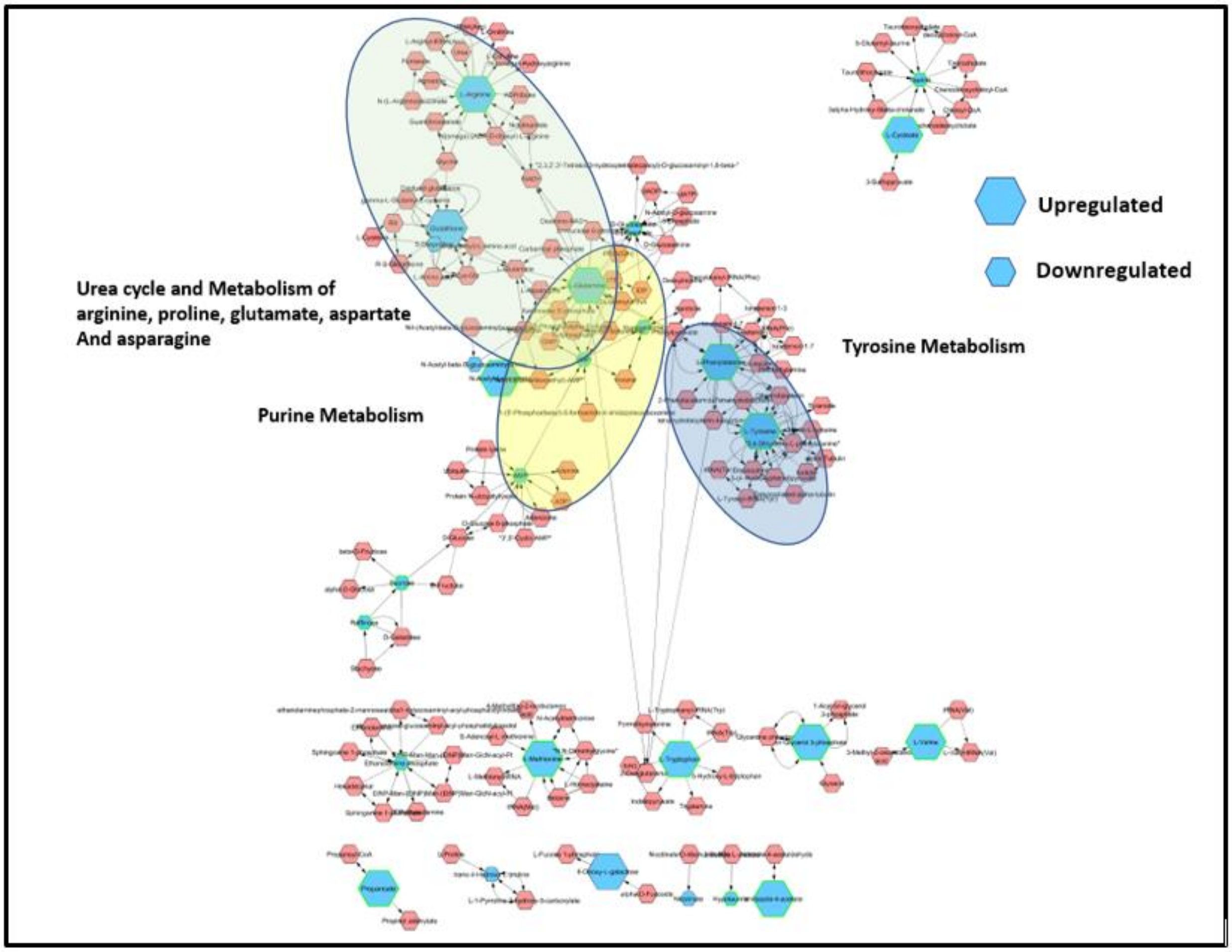

Figure S15, Cytoscape-based pathway analysis using HILIC-MS data showing several metabolic pathways that differ between veteran sarcoidosis and civilian sarcoidosis cohorts.
